# Whole-exome sequencing revealed a novel mutation of the *ALMS1* gene in a Chinese family with Alström syndrome: a case report

**DOI:** 10.1186/s12887-024-04949-y

**Published:** 2024-08-02

**Authors:** Ming Hu, Shuang Chen, Jinyuan Wu, Rong Wang

**Affiliations:** 1 Department of Otorhinolaryngology Head and Neck Surgery, Tianjin First Central Hospital, Tianjin, 300192 China; 2 Key Laboratory of Auditory Speech and Balance Medicine, Tianjin, 300192 China; 3 Key Medical Discipline of Tianjin (Otolaryngology), Tianjin, 300192 China; 4 Department of Laboratory Medicine, School of Medical Technology, Tianjin Medical University, Tianjin, 300203 China; 5 Department of Ophthalmology, Tianjin First Central Hospital, Tianjin, 300192 China

**Keywords:** Alström syndrome, Whole exome sequencing, *ALMS1*, Mutation

## Abstract

**Background:**

Alström syndrome (AS) is a rare autosomal recessive disorder that leads to multiple organ fibrosis and failure. Precise diagnosis from the clinical symptoms is challenging due to its highly variabilities and its frequent confusion with other ciliopathies and genetic diseases. Currently, mutations in the *ALMS1* gene have been reported as a major cause of AS, thus, it is crucial to focus on the detection and discovery of *ALMS1* mutations.

**Case presentation:**

We present a case of a 13-year-old Chinese boy weighing 70 kg and standing 168 cm tall. He has two younger brothers. Their parents hail from different ancestral homes in eastern and northern China. The patient’s primary clinical findings included visual impairment at the age of four and progressive hearing loss starting at the age of ten. Subsequently, at the age of twelve, the patient developed hyperlipidaemia and hyperinsulinemia. Ultrasonographic findings indicated the presence of gallstones and mild fatty liver. His Body Mass Index (BMI) significantly increased to 25 kg/m^2^ (ref: 18.5–23.9 kg/m^2^). Additionally, echocardiography revealed mild mitral and tricuspid regurgitation. Ultimately, Whole Exome Sequencing (WES) identified a new missense mutation in the *ALMS1* gene (NG_011690.1 (NM_015120): c.9536G > A (p.R3179Q)). This missense mutation generated an aberrant splicer and disrupted the stability and hydrophobicity of proteins, which preliminarily determined as “ likely pathogenic”. Therefore, considering all the above symptoms and molecular analysis, we deduced that the patient was diagnosed with AS according to the guidelines. We recommended that he continue wearing glasses and undergo an annual physical examination.

**Conclusion:**

In this case report, we report a novel homozygous *ALMS1* mutation associated with AS in the Chinese population, which expands the mutation spectrum of *ALMS1*. Genetic testing indeed should be incorporated into the diagnosis of syndromic deafness, as it can help avoid misdiagnoses of AS. While there is no specific treatment for AS, early diagnosis and intervention can alleviate the progression of some symptoms and improve patients’ quality of life.

**Supplementary Information:**

The online version contains supplementary material available at 10.1186/s12887-024-04949-y.

## Background

Alström syndrome (AS, ALMS; OMIM#203,800) is an autosomal recessive inherited disease that was first described by Carl Henry Alström of Sweden in 1959 [1]. The incidence rate of AS is approximately 1/million to 9/million [2], with no sex differences, and the incidence rate significantly increases in offspring from inbreeding. AS has been described as a syndrome [3, 4] characterized by obesity, type 2 diabetes mellitus (T2DM) [2], and retinal and cochlear degeneration that progresses as patients age and other multiple organ failures occur [5].

Among these symptoms, progressive visual impairment and sensorineural deafness [6] are the most consistent symptoms [3]. Notably, deafness occurs in 70% of patients within the first 10 years after birth and may develop into moderately severe hearing loss or deafness within 10 ~ 20 years [7]. However, diagnosing AS can be challenging due to its progressive primary and secondary symptoms, which are often confused with other ciliopathies and genetic disorders, such as Bardet-Biedl Syndrome [8], idiopathic cardiomyopathy [9], and Leber congenital amaurosis [10].

Currently, in addition to clinical symptoms, one of the “gold standards” for diagnosing AS is the presence of an *ALMS1* mutation [11]. A double allele mutation in the *ALMS1* gene leading to AS was first discovered by Collin, G.B., et al. in 2002 [12]. *ALMS1*, located on chromosome 2p13, spans 23 exons and encodes a predicted 461.2-kDa protein of 4,169 amino acids (AA) [13]. Hearn et al. studied the subcellular localization and tissue distribution of *ALMS1* through immunofluorescence and reported that *ALMS1* is widely expressed and located at the base of centrosomes and cilia [14]. *ALMS1* has been implicated in the function, formation, and/or maintenance of primary cilia (PCs). PCs play a crucial role in mechanical and chemical sensory perception, and their dysfunction is associated with developmental disorders and severe diseases [15].

Neurogenic deafness and visual impairment are the most consistent symptoms of AS, and the function of the *ALMS1* gene in auditory and visual maintenance has also been validated in some animal models. The ultrastructural abnormalities of the three-dimensional ciliary bundles in the outer hair cells of the P22 *ALMS1* ^− / −^ mouse cochlea are consistent with those in the P2 cochlea, indicating that the defect is preserved until maturity [16]. In *ALMS1* ^− / −^ mice, the transport of 11-cis retinol to the outer ganglia may be promoted, thereby enhancing the turnover of chromophores in cone cells and leading to the deterioration of cone cells in AS [17].

AS results from biallelic homozygous or compound heterozygous mutations. The *ALMS1* gene mutations are mostly found in the hot-spot exons 8, 10, and 16 [13, 18, 19]. However, the distribution of these mutations varies among different races. The c.10775delC (p. Thr3592 Lysfs*6) mutation in exon 16, found in individuals of British descent, is the most frequently reported [20]. Ozanturk A et al. evaluated mutations in *ALMS1* in 61 Turkish patients and reported a total of 20 different nucleotide changes [21]. In this report, we present a novel molecular finding in a Chinese family, underscoring the importance of preemptive genetic screening and diagnosis for AS.

## Methods

### Clinical features

The patient is a 13-year-old boy born to non-consanguineous Chinese parents. He has two younger brothers. He experienced blurred vision at the age of 4 and started wearing glasses at the age of 5. In 2018, the patient began to experience photophobia at the age of 10 and had difficulty navigating stairs due to frequent falls.

### Hearing tests

At the age of ten, the patient underwent an Auditory Brainstem Response (ABR) test [22], a 40 Hz Auditory Event-Related Potential (40 Hz AERP) test [23] and a Distortion Product Otoacoustic Emission (DPOAE) test [24]. These tests were repeated three years later. Additionally, a caloric test was conducted to induce and observe vestibular responses by stimulating the semicircular canal with temperature.

### Visual acuity examination

The patient underwent Optical Coherence Tomography (OCT) [25] examination of the macula in 2018 and 2021. In 2021, he also underwent visual field defect detection and an Electroretinogram (ERG) [26] test.

### Imaging detection

The patient underwent ultrasonic examinations of the liver, spleen, and kidney at the ages of 12 and 13. Additional ultrasonic examinations of the gallbladder and pancreas, spinal radiology, and cardiac echocardiography were conducted when he was 13 years old.

### Biochemical tests and BMI records

When the patient was 12 years old, blood was drawn for biochemical tests, including of insulin, C-peptide, Alanine aminotransferase (ALT), Alkaline phosphatase (ALP), r-glutamyltranspeptidase (r-GT), Leucine aminopeptidase (LAP), Adenosine deaminase (ADA), Aspartate aminotransferase (AST), AST mitochondrial isoenzyme (m-AST), Lactate dehydrogenase (LDH), α-hydroxybutyrate dehydrogenase (HBDH), Creatine kinase (CK),Creatine kinase isoenzyme (CKMB), Triglyceride (TG), Total cholesterol (TCho), High-density lipoprotein cholesterol (HDL-C), Low density lipoprotein cholesterol (LDL-C), Apolipoprotein A-1 (APOA1), Apolipoprotein B (APOB), and α1-antitrypsin (AAT). The patient’s BMI was calculated and recorded by his parents from the age of 10 to 13.

### Gene detection and analysis

The proband’s genomic DNA was extracted from peripheral blood collected in EDTA anticoagulant tubes and sent to MyGenostics, Beijing, for mutation identification using Whole Exome Sequencing (WES). The sequence reads were aligned to the human reference genome (hg19), and Sanger sequencing was performed to confirm the mutations.

The splicing effects of the novel mutations were analysis using online databases, including Predictions from BDGP (http://www.fruitfly.org/seq_tools/splice.html), ASSP (http://wangcomputing.com/assp/index.html), and NetGene2 (http://www.cbs.dtu.dk/services/NetGene2/). The physical and chemical properties of the proteins were analysed via the ProtScale (https://web.expasy.org/protscale/) and PolyPhen-2 (http://genetics.bwh.harvard.edu/pph2/) databases.

## Results

**Fig. 1:**
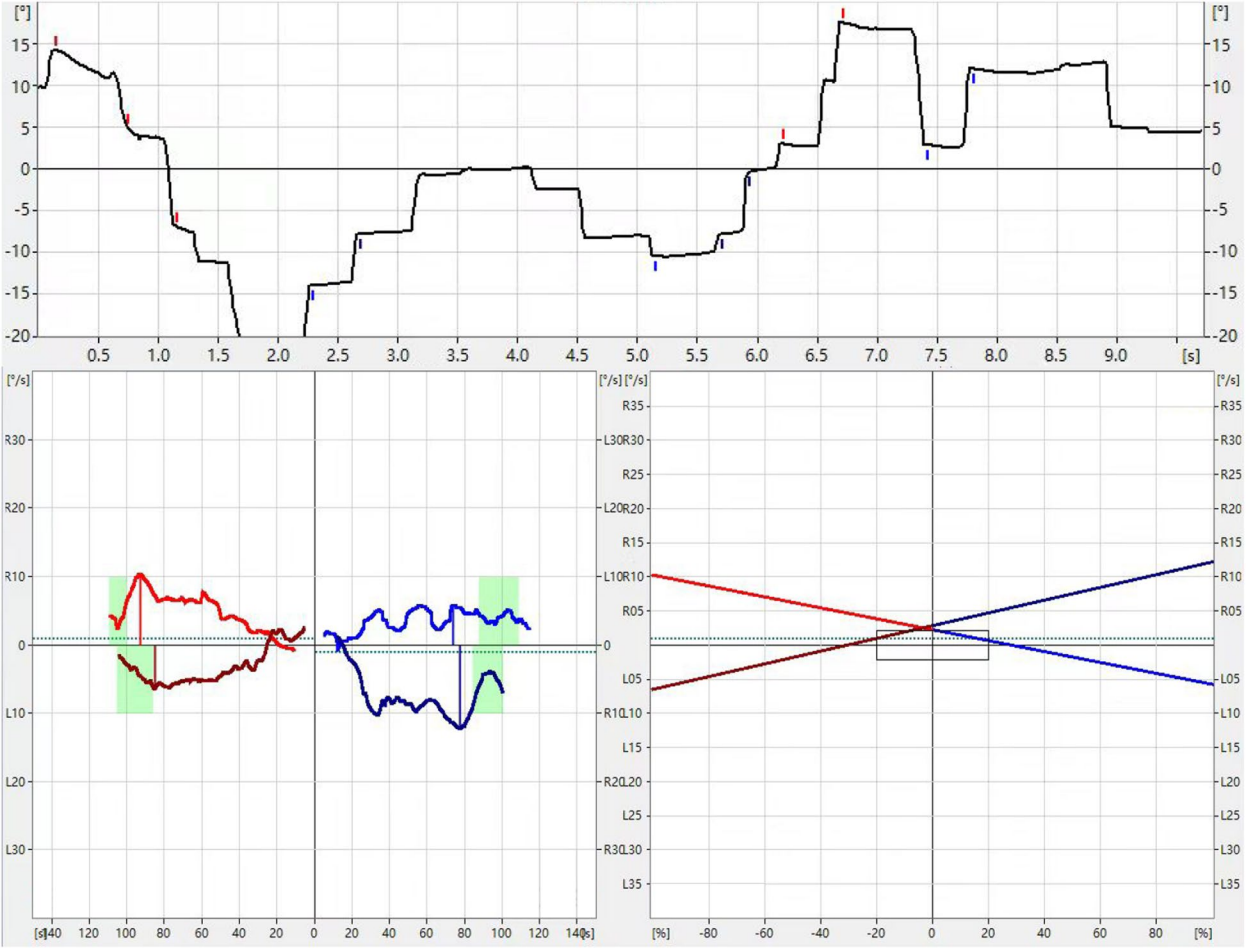
Videonystagmography results

In terms of auditory function, Table 1 shows that the patient’s hearing capability gradually deteriorated, with binaural hearing becoming significantly worse by the age of 13, no significant DPOAE was detected in either ear at any frequency. Additionally, Videonystagmography (VNG) revealed that the low-frequency functional response of the bilateral horizontal semicircular canals was normal, with a rightward nystagmus advantage (Fig. 1).

**Table 1:** Response threshold of the hearing test

Items	Left ear		Right ear	
	2018 (10yrs)	2021 (13yrs)	2018 (10yrs)	2021 (13yrs)
ABR	35 dbnHl	70 dBnHL	30 dbnHl	65 dBnHL
40 Hz AERP	30 dbnHl	20 dBnHL	20 dbnHl	20 dBnHL

**Fig. 2:**
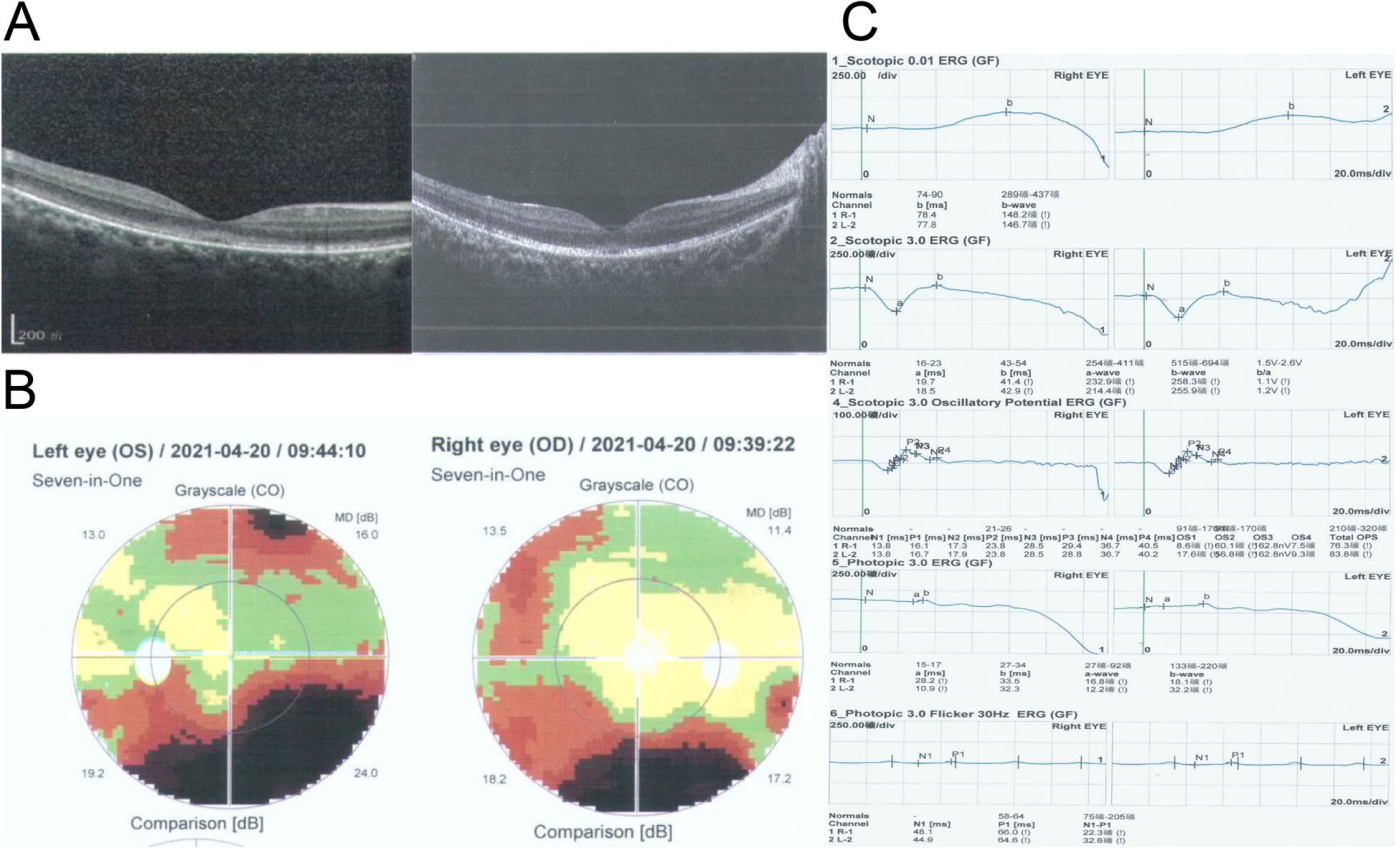
Visual examination. **A** Optical Coherence Tomography (OCT) of the proband showing thinning and irregular arrangement of retinal pigmentation. The left figure displays the results from 2017, while the right figure presents the results from 2021. **B** Visual field examination revealed visual field defects in both eyes. **C** Results of the Electroretinogram (ERG)

Visual examination results indicated that the patient developed nystagmus and photophobia at the age of 10. As the patient aged to 13 years, a thinned Retinal Pigment Epithelium (RPE) and irregular arrangement of the retina were observed (Fig. 2A). Visual field examination revealed visual field defects in both eyes, with the left eye being more severely affected (Fig. 2B). From Fig. 2C, ERG scotopic rods showed a decrease in b-wave amplitude, the maximum response a-wave amplitude was generally normal, the maximum response b-wave amplitude was moderately reduced, the ops response wave amplitude was moderately reduced, and the cone response b-wave and 30 Hz response wave amplitude of rods were severely reduced.

**Fig. 3:**
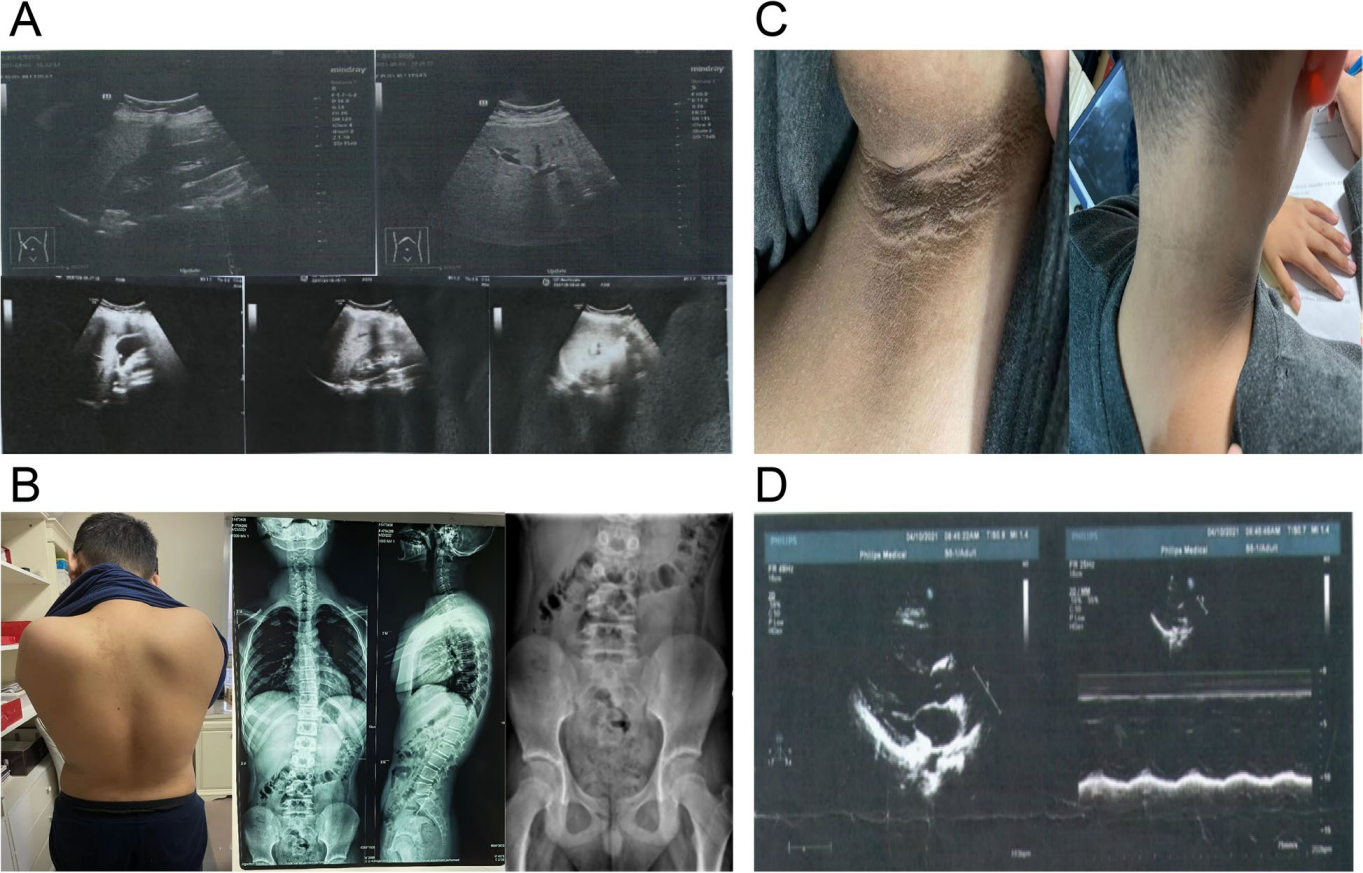
Ultrasonic examination and echocardiography. **A** Results of ultrasound examinations. The two images at the top are from 2021, and the three images at the bottom are from 2022. **B** Development of scoliosis and epiphysis. **C** Presence of acanthosis nigricans. **D** Echocardiography findings

Ultrasound of the patient at 12 years old showed uneven enhancement of parenchymal echoes, suggesting uneven fatty liver. Spleen and renal ultrasound showed no abnormalities in size and morphology. However, at the age of 13, ultrasonography of the liver revealed a strong echo mass measuring 5 × 4 mm in the gallbladder cavity, with sound and shadow visible behind, suggesting gallstones and mild fatty liver (Fig. 3A), while the pancreas, kidneys, and spleen appeared normal. The anterior posterior bitmap of the full length of the spine showed that the severity of the patient’s scoliosis was grade 5 according to the Risser sign and scale closure (Fig. 3B). Systemic examination revealed acanthosis nigricans (Fig. 3C). Echocardiography suggested mild MR and TR (Fig. 3D).

The biochemical test results showed that C-PEP, ALT, ALP, r-GT, AD, AST, m-AST, LDH, CK, CK-MB, TG, TCho, APOA1, APOB and AAT were normal, but the following indicators were abnormal: LAP 73 U/L (ref: 30–70 U/L). HBDH 194 U/L (ref: 72–182 U/L). HDL-C was 0.96 (ref: > 1.45 mmol/L). LDL was 2.72 mmol/L (ref: 0–2.59 mmol/L). His insulin concentration was 219.8 pmol/L (ref: 17.8–173 pmol/L). Elevated LAP and LDL-C, as well as decreased HLDL-C, suggest hyperlipidaemia in patients. Both CK and CKMB were normal, with only a slight increase in HBDH, indicating that the patient may be in an infected state. Furthermore, increased insulin (219.8 pmol/L, ref: 17.8–173 pmol/L) suggested hyperinsulinemia. Meanwhile, the patient present IGT (Impaired glucose tolerance), according to the lower FBG (Fasting blood glucose) with 4.88 mmol/L (ref: < 7 mmol/L) and higher PBG (Postprandial blood glucose) with 8.26 mmol/L (ref: > 7.8 mmol/L and < 11.1 mmol/L). Additionally, the patient’s BMI significantly increased from 23.4 kg/m^2^ at 10 years of age to 25 kg/m^2^ (ref: 18.5–23.9) at 12 years of age.

**Fig. 4:**
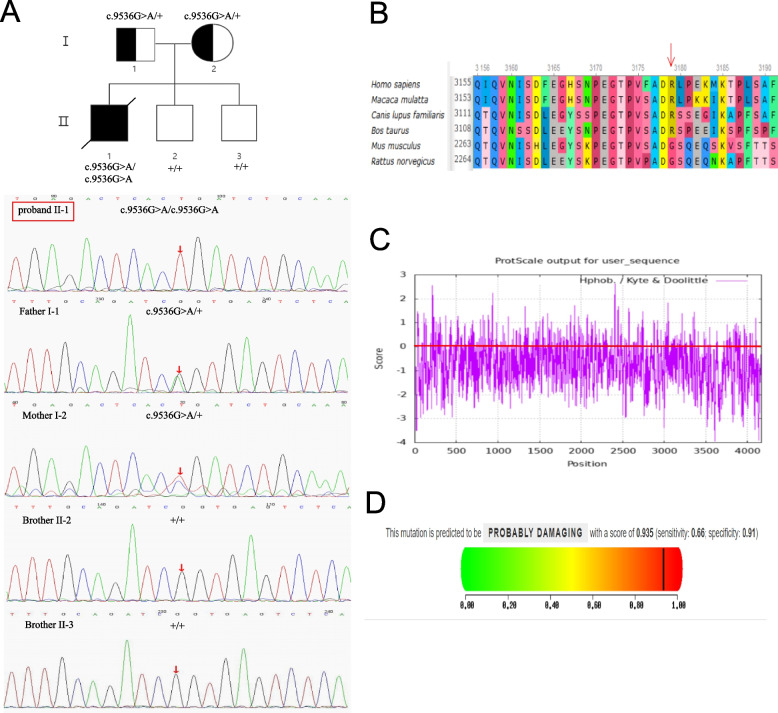
Molecular detection. **A** The pedigree of this family. The proband (II-1), parents (I-1, I-2), and two brothers (II-2, II-3), along with the sequencing results of the *ALMS1* family. **B** Position of the amino acid mutation. **C** The abscissa represents the sequence position, and the ordinate represents the amino acid scale value. The scale is defined by the high scoring value of hydrophobic amino acids: > 0 indicates hydrophobicity, and < 0 indicates hydrophilicity. The negative value predicted from the graph combined with gravy suggests that the protein produced by mutation sites is hydrophilic. **D** The closer the score is to 1, the greater the potential damage. The results are categorized into beneficial, probably damaging, and possibly damaging

Both WES and Sanger sequencing revealed that the patient had a novel mutation in exon 10 of the *ALMS1* gene, NG_011690.1 (NM_015120): c.9536G > A, on chromosome 2. Since the patient’s parents and two brothers did not present any clinical symptoms of AS, Sanger sequencing was performed on his family members. The results showed that both his father and mother were carriers of the pathogenic variant (Fig. 4A). However, neither of his brothers carried the pathogenic variant, leading to the speculation that the patient’s pathogenic variant was recessively inherited from both his parents. As an assumption, the genetic pattern of pathogenic genes can be seen from the genetic map of the family (Fig. 4A). Therefore, according to the updated ACMG guidelines [22], the variation in the *ALMS1* gene NG_011690.1 (NM_015120), c.9536 G > A (p.R3179Q) is preliminarily determined as "likely pathogenic", PM2 + PM3_Supporting (HOM) + PP3 + PP4_Strong + PP1_Supporting.

Indeed, all three online databases showed that *ALMS1* c.9536 G > A has undergone variable splicing. Furthermore, this missense mutation led to the change of arginine at position 3179 to glutamine (Fig. 4B). The ProtScale database predicts that the hydrophobicity scales of amino acids at the mutation site will change from -4.500 to -3.500 (Fig. 4C). Consequently, altered protein structure may influence the normal function of *ALMS1*. Analysis of the PolyPhen-2 database revealed that the C.9536 g > A (p.R3179Q) mutation is likely to cause disease (Fig. 4D).

## Discussion

Considering all the above clinical findings and in accordance with the “Consensus Clinical Management Guidelines for Alström syndrome” [27], we deduced that the patient had a diagnosis of AS (Table 2). First, retinal dystrophy is a major clinical manifestation of AS. Next, hearing impairment is also very common in AS patients and usually manifests as progressive bilateral sensorineural hearing loss. Approximately 70% of children experience hearing impairment before the age of 10 years. Additionally, almost all AS patients have hyperinsulinemia, ultimately leading to insulin resistance. Recent studies [28] have shown that the C-peptide level is an important index of early-onset type 2 diabetes patients, and the frequency of *ALMS1* pathogenic mutations in early-onset type 2 diabetes patients with insulin resistance is greater than that in other groups, but the median age of onset was 16 years old. This is consistent with our case in which the C-peptide level was 1.16 nmol/L (ref: 0.37–1.47 nmol/L) at the patient’s age of 12, which is close to the upper limit. Furthermore, it has been reported that most AS patients experience dyslipidemia. As to BMI, most of AS patients appear obesity with relatively higher BMI, while it will decrease in the adult according to the guideline [27, 29]. Approximately 68% of AS patients have thoracolumbar scoliosis, kyphosis, or lordosis [30]. Finally, according to AS guidelines, the diagnosis of AS is mainly based on clinical symptoms that gradually increase with age. AS is an autosomal recessive genetic disease, and genetic testing has revealed that pathogenic variations in *ALMS1* are more important for confirming its diagnosis. Indeed, both the patient and his parents carried the *ALMS1* mutation in this case. Collectively, our patient’s clinical phenotype was consistent with the above conclusion.

**Table 2:** Evidence levels and strength of AS diagnosis and management

Items	Evidence levels	Strength of recommendation
Hearing lost gradually increased	A*	Strong
Nystagmus and photophoria; Thinning and irregular arrangement of retinal pigmentation; visual field defect; cone rod dystrophy	B*	Strong
Hyperlipidemia; fasting insulin level increased	B*	Strong
Gallstone and mild fatty liver	B*	Strong
Mild mitral and tricuspid regurgitation	C*	Strong
Mutation on ALMS1 gene	A*	Strong

A*. High-quality evidence. ﻿B*. Moderate-quality evidence ﻿C*. Low-quality evidence

Certainly, since many *ALMS1* mutations have been reported currently, researchers are curious whether there is any relationship between the spectrum of clinical features and variants. However, as Ayşegül Ozantürk’s group found [21], although all AS patients exhibit progressive multisystem involvement characterized by neurosensory degeneration and metabolic defects, the mutation type is not directly related to the clinical phenotype. Furthermore, research by Natascia Tahani [31] and You Wang [32] revealed that clinical manifestations vary with age. Typically, infants present with cone-rod cell malnutrition accompanied by nystagmus and severe visual impairment [31]. Childhood often involves retinal cone-rod cell malnutrition, hearing loss, and insulin resistance. Adolescents may experience diabetes and hypertriglyceridemia, while adults face progressive liver and kidney dysfunction, metabolic fatty liver disease, scoliosis, and various endocrine and metabolic disorders. Additionally, a study [33] suggested that the same gene mutation can lead to vastly different clinical characteristics, onset times, and severity—even within the same family. These variations may be influenced by various potential genetic modification factors (such as the environment and infectious exposure).

To date, there is no specific therapy for ALMS mutations, and the mainstay of management involves multidisciplinary and multidisciplinary teams of experts, as early diagnosis and intervention can slow the progression of multiorgan dysfunctions and improve the longevity and quality of life of patients. Cochlear implants can be used to correct hearing loss [34]. Skeletal complications, usually scoliosis, require adequate physical therapy. Weight loss through appropriate lifestyle adjustments, such as a low-calorie, low-fat diet and regular physical activity, is likely to improve blood sugar and lipid levels [35], thereby reducing the risk of type 2 diabetes, metabolic syndrome, and cardiovascular disease. In addition, some symptoms can be controlled with medication. Since the patient in our case did not show severe symptoms, we only asked him to continue wearing glasses to improve his vision and to follow up regularly.

## Conclusion

In this case, we identified a novel homozygous pathogenic variant in *ALMS1* previously unreported in a Chinese population. It is worth noting that WES is particularly useful for the accurate and early diagnosis of diseases with progressive symptoms, such as AS, which is crucial for effective patient management. Once the patient’s genetic diagnosis is confirmed, testing the corresponding loci in their relatives can aid in diagnosis or carrier screening and is beneficial for providing genetic counselling to the proband’s parents regarding reproduction.

### Supplementary Information

Supplementary Material 1.

## Data Availability

DNA sequence data that support the findings of this study have been deposited in the National Center for Biotechnology Information with the primary accession code 7840 (ALMS1, NCBI ID: 7840, DNA sequence was listed in the Supplementary file 1). Protein sequence data that support the findings of this study have been deposited in the Universal Protein with the primary accession code Q8TCU4 (ALMS1, Uniprot ID: Q8TCU4, Protein sequence was listed in the Supplementary file 1). Whole exome sequencing data are available at the following URL: http://ns.mygeno.cn:5000/sharing/It2nuyoly, and password is 123.

## Abbreviations

AS: Alström Syndrome
WES: Whole Exome Sequencing
40 Hz AERP: 40 Hz Audit Event Related Potentials
ABR: Auditory Brainstem Response
DPOAE: Distortion Product Otoacoustic Emission
OCT: Optical Coherence Tomography
ERG: Electroretinogram
T2DM: Type 2 Diabetes
NAFLD: Nonalcoholic Fatty Liver Disease
PC: Primary Cilia
AA: Amino Acids
VNG: Videonystagmography
BMI: Body Mass Index
RPE: Retinal Pigment Epithelium
TRD: Tandem Repeat Domain
OHC: Outer Hair Cells
