# Supplementary material for: Whole-exome sequencing revealed a novel mutation of the ALMS1 gene in a Chinese family with Alström syndrome: a case report
Source: BMC Pediatr. 2024 Aug 2;24:494. doi: 10.1186/s12887-024-04949-y (PMC11295688; doi:10.1186/s12887-024-04949-y)
Supplement: Supplementary file 1 — Supplementary Material 1. [file 12887_2024_4949_MOESM1_ESM.docx]

Supplementary file 1:

**1. DNA sequencing**

**WT-** **DNA sequencing data** **(Gene : ALMS1, NCBI ID :7840 )：**

AGGCGGGCGGCACTGCGCCTAAGCTGGGCCACAACCGCCAGTCAGGGCTCTCCCCTTCCCCTCCCTCCCCCCCTCCTCCTCCTCCTCTGCCGCCCAGAGCGAGACACCAACATGGAGCCCGAGGATCTGCCATGGCCGGGCGAGCTGGAGGAGGAGGAGGAGGAGGAGGAGGAGGAGGAGGAGGAGGAAGAGGAGGAGGCTGCAGCGGCGGCGGCGGCGAACGTGGACGACGTAGTGGTCGTGGAGGAGGTGGAGGAAGAGGCGGGGCGGGAGTTGGACTCCGACTCTCACTACGGGCCCCAGCATCTGGAAAGTATAGACGACGAGGAGGACGAGGAGGCCAAGGCCTGGCTGCAGGCGCACCCCGGCAGGATTTTGCCTCCGCTGTCGCCCCCGCAGCACCGCTACTCGGAGGGCGAGCGGACCTCCCTGGAGAAGATTGTTCCATTGACCTGTCATGTATGGCAACAGATAGTATATCAAGGCAATAGTAGAACACAAATTTCTGATACTAATGTGGTCTGTTTGGAAACAACAGCTCAGCGGGGTTCTGGGGATGATCAGAAAACAGAATCTTGGCATTGTCTTCCTCAAGAAATGGACTCTTCCCAAACCTTGGATACATCCCAGACTAGGTTTAATGTGAGAACGGAAGATACTGAAGTGACAGACTTCCCCTCTCTGGAGGAGGGCATATTGACGCAATCAGAAAATCAAGTAAAGGAACCCAACAGAGATCTCTTCTGTTCTCCACTGCTAGTCATACAAGATAGCTTTGCTTCTCCTGATTTGCCTTTGCTGACCTGTTTGACACAAGACCAAGAATTTGCGCCTGATTCTTTATTTCATCAAAGTGAACTAAGTTTTGCACCTCTGAGGGGAATTCCTGATAAGTCTGAAGATACTGAATGGTCTTCTCGACCATCGGAAGTTAGTGAAGCTTTATTCCAGGCTACTGCAGAAGTAGCTTCAGACTTAGCAAGCAGTCGCTTTAGTGTATCTCAGCACCCGCTTATAGGCAGCACAGCTGTTGGGTCTCAGTGCCCTTTTTTACCTTCTGAACAAGGGAATAATGAAGAGACTATTTCGTCTGTTGATGAACTGAAAATTCCCAAAGACTGTGATCGTTATGATGATCTTTGTTCATATATGTCATGGAAGACACGAAAAGATACACAGTGGCCTGAAAACAATTTAGCTGATAAAGATCAAGTTTCAGTTGCAACTTCATTTGACATAACTGATGAAAACATAGCTACTAAAAGAAGTGACCATTTTGATGCTGCTCGTTCATATGGGCAGTATTGGACACAGGAAGATTCATCTAAGCAGGCAGAAACATATTTAACCAAGGGCCTGCAGGGGAAGGTTGAGTCTGACGTCATTACTCTGGATGGCCTAAATGAAAATGCTGTTGTATGCAGTGAAAGAGTTGCTGAACTACAAAGAAAGCCAACAAGAGAGTCGGAATATCACTCTTCAGATCTCAGAATGTTGAGGATGTCTCCTGACACTGTGCCAAAGGCTCCTAAACATTTAAAAGCAGGAGACACTTCTAAAGGAGGCATAGCTAAAGTTACTCAATCCAACTTGAAGTCAGGCATCACTACCACTCCTGTTGATTCAGACATTGGATCTCATTTATCCTTGTCCCTTGAGGACCTGTCTCAGTTGGCTGTAAGTTCTCCTCTAGAAACTACTACTGGTCAACACACTGATACTCTCAACCAAAAGACATTAGCAGATACTCATCTAACTGAAGAGACTCTGAAAGTCACAGCTATTCCTGAACCAGCTGACCAGAAGACTGCAACACCAACAGTACTCTCTAGTTCCCACTCACATAGGGGGAAGCCCAGCATTTTCTACCAGCAGGGCTTGCCAGACAGTCATCTAACTGAAGAGGCTTTGAAAGTTTCAGCTGCTCCTGGACTAGCTGACCAGACAACTGGCATGTCAACTCTAACCTCTACTTCCTACTCACATAGAGAGAAGCCTGGTACTTTTTACCAACAAGAGTTACCAGAGAGTAACTTAACCGAAGAGCCTTTGGAAGTTTCAGCTGCTCCTGGCCCAGTGGAGCAGAAGACGGGAATACCTACAGTATCCTCTACATCCCACTCACATGTAGAGGACCTCCTCTTTTTCTATCGACAGACCTTGCCAGATGGTCATCTAACTGATCAGGCTCTGAAAGTCTCAGCTGTGTCTGGACCAGCTGACCAGAAGACTGGGACAGCAACAGTACTCTCTACTCCCCACTCACATAGAGAGAAGCCTGGTATTTTTTACCAACAAGAGTTCGCAGACAGTCATCAAACTGAAGAGACTCTTACTAAAGTTTCAGCCACTCCTGGACCAGCTGACCAGAAGACTGAGATACCAGCAGTACAGTCTAGTTCTTACTCACAAAGAGAAAAGCCTAGTATTTTGTACCCACAGGACTTAGCAGACAGTCATCTACCTGAAGAGGGTCTGAAAGTTTCAGCTGTTGCTGGACCAGCTGACCAGAAGACTGGCCTACCAACAGTACCCTCTAGTGCATACTCACACAGAGAGAAGCTCCTTGTTTTCTACCAACAGGCCTTGCTGGACAGCCATCTACCCGAAGAGGCTCTGAAAGTTTCAGCTGTTTCTGGACCAGCTGACGGAAAGACTGGGACACCAGCTGTAACCTCTACTTCCTCTGCGTCCTCTTCACTTGGAGAAAAGCCCAGTGCTTTCTATCAGCAGACCTTACCCAATAGTCATCTAACTGAAGAGGCTCTGAAAGTATCAATTGTTCCTGGACCAGGTGATCAGAAGACTGGGATACCCTCAGCACCATCTAGTTTCTACTCACACAGAGAGAAGCCCATTATTTTTTCCCAGCAGACCCTGCCAGACTTTCTTTTCCCTGAAGAAGCTCTGAAGGTTTCAGCTGTTTCTGTATTGGCTGCCCAGAAGACTGGGACACCAACAGTGTCCTCTAATTCTCACTCACATAGCGAGAAATCTAGTGTTTTCTACCAGCAAGAGTTGCCAGACAGTGATCTACCTAGAGAATCTCTGAAAATGTCTGCTATTCCTGGACTGACTGACCAGAAGACTGTCCCAACACCAACAGTACCTTCAGGTTCCTTCTCACATAGAGAGAAGCCCAGTATTTTCTATCAACAGGAGTGGCCAGATAGTTATGCAACTGAAAAGGCTCTGAAAGTTTCAACTGGCCCTGGACCAGCTGACCAGAAGACTGAGATACCAGCAGTACAGTCTAGTTCTTACCCACAGAGGGAGAAGCCTAGTGTTTTGTACCCACAGGTGTTATCAGACAGTCATCTACCTGAAGAGAGTCTGAAAGTTTCAGCCTTCCCTGGACCAGCTGACCAGATGACTGACACACCAGCAGTACCGTCTACTTTCTACTCACAAAGAGAGAAGCCTGGTATTTTCTACCAACAGACCTTGCCAGAGAGTCATCTGCCTAAAGAGGCTCTGAAAATTTCAGTAGCTCCTGGACTAGCAGACCAGAAGACTGGCACACCAACTGTAACCTCAACTTCCTACTCACAACATAGAGAAAAGCCCAGCATTTTCCACCAGCAGGCCTTGCCAGGTACTCATATACCTGAAGAGGCTCAGAAAGTTTCAGCTGTTACTGGACCAGGTAACCAGAAGACTTGGATACCAAGAGTACTTTCTACCTTCTACTCACAAAGAGAGAAACCTGGTATTTTCTATCAACAGACCTTGCCAGGTAGTCACATACCTGAAGAGGCACAGAAAGTTTCACCTGTTCTTGGACCAGCTGACCAGAAGACTGGGACACCAACTCCAACCTCTGCTTCTTACTCACACACAGAGAAGCCTGGTATTTTCTACCAACAGGTCTTGCCAGATAATCATCCAACTGAAGAGGCTCTGAAAATTTCAGTTGCCTCTGAACCAGTTGACCAGACAACTGGCACACCAGCTGTAACCTCTACTTCCTACTCACAATATAGAGAGAAGCCCAGCATTTTCTACCAACAGTCGTTGCCAAGTAGTCATCTAACTGAAGAGGCTAAGAATGTTTCAGCGGTTCCTGGACCAGCTGACCAGAAGACTGTGATACCAATTTTACCCTCTACTTTCTACTCACACACAGAGAAGCCTGGTGTTTTCTACCAACAGGTCTTGCCACATAGTCATCCAACTGAAGAGGCTCTGAAAATTTCAGTTGCCTCTGAACCAGTTGACCAGACAACTGGCACACCAACTGTAACCTCTACTTCTTACTCACAACATACAGAGAAGCCGAGTATTTTCTACCAACAGTCGTTGCCAGGTAGTCATCTAACTGAAGAGGCTAAGAACGTTTCAGCGGTTCCTGGACCAGGTGACCGGAAGACTGGGATACCAACTTTACCCTCTACTTTCTACTCACACACAGAGAAGCCTGGTAGTTTCTACCAACAGGTCTTGCCACATAGTCATCTACCTGAAGAGGCTTTGGAAGTTTCAGTTGCTCCTGGACCAGTTGACCAGACGATTGGCACACCAACTGTAACCTCCCCTTCCAGCTCATTTGGAGAGAAGCCCATTGTTATCTACAAACAGGCCTTTCCAGAGGGTCATCTACCTGAAGAGTCTCTGAAAGTTTCAGTTGCTCCTGGACCAGTTGGCCAGACAACTGGCGCACCAACTATAACCTCTCCTTCCTACTCACAACATAGAGCAAAGTCTGGCAGTTTCTACCAACTGGCATTGCTAGGTAGTCAAATACCTGAAGAGGCTCTCAGAGTTTCTTCTGCTCCTGGACCAGCTGACCAGACAACTGGCATACCAACCATAACCTCTACTTCCTACTCATTTGGAGAGAAGCCGATTGTTAACTACAAACAGGCCTTTCCAGATGGTCATCTACCTGAAGAGGCTCTGAAAGTTTCCATTGTTTCTGGACCTACTGAAAAAAAGACTGACATACCAGCAGGACCTTTAGGTTCCAGTGCACTTGGAGAGAAGCCCATTACTTTCTACCGGCAGGCTCTGCTAGACAGTCCTCTAAATAAAGAGGTTGTGAAAGTTTCAGCTGCTCCTGGACCAGCTGACCAGAAGACTGAGACATTACCAGTACATTCTACTAGCTACTCAAATAGGGGGAAGCCTGTCATTTTCTACCAGCAGACCCTATCAGACAGTCATTTACCTGAAGAAGCTCTGAAAGTTCCACCTGTTCCTGGACCAGATGCCCAGAAGACTGAGACACCATCAGTATCCTCTAGTTTATACTCATATAGAGAGAAGCCCATTGTCTTCTACCAACAGGCCCTGCCAGACAGTGAGCTAACTCAAGAAGCTCTGAAAGTTTCAGCTGTTCCTCAACCAGCTGACCAGAAGACTGGGTTATCTACTGTAACTTCCTCTTTCTATTCACATACAGAGAAGCCTAATATTTCTTACCAGCAAGAGTTGCCAGATAGTCATCTAACTGAAGAGGCTCTGAAAGTTTCAAATGTTCCTGGACCAGCTGACCAGAAGACTGGGGTATCAACAGTAACCTCTACTTCCTACTCACACAGAGAGAAGCCCATTGTTTCCTACCAGCGAGAGTTGCCGCATTTTACTGAAGCAGGTTTGAAAATTTTAAGAGTTCCTGGACCAGCTGACCAGAAGACTGGAATAAACATCCTGCCCTCTAATTCCTACCCACAGAGAGAGCACTCTGTCATTTCTTATGAGCAGGAGTTGCCAGATCTTACTGAAGTAACTTTGAAAGCAATAGGGGTTCCTGGGCCTGCTGACCAGAAGACTGGGATACAAATAGCATCCTCTAGTTCCTACTCAAATAGAGAGAAGGCCAGTATTTTTCATCAGCAGGAGTTGCCAGATGTTACTGAAGAAGCTTTAAATGTTTTTGTTGTTCCTGGACAAGGTGACCGGAAGACTGAGATACCAACAGTACCTTTAAGTTACTACTCACGTAGAGAGAAGCCCAGTGTTATCTCTCAACAGGAGTTGCCAGACAGTCATCTCACAGAAGAGGCTCTGAAAGTTTCACCTGTTTCTATACCAGCAGAGCAGAAGACTGGGATACCAATAGGACTGTCTAGTTCCTACTCACATTCACATAAAGAGAAACTCAAGATTTCAACTGTGCATATACCAGATGACCAGAAAACTGAGTTTCCAGCAGCTACCCTTAGTTCCTACTCACAAATAGAGAAGCCCAAGATTTCAACTGTGATTGGACCAAATGACCAGAAGACTCCATCCCAGACAGCTTTTCATAGTTCCTATTCTCAAACAGTAAAGCCCAATATTTTATTTCAACAGCAGTTGCCAGATAGAGATCAAAGTAAAGGTATTCTAAAGATTTCAGCTGTCCCTGAACTAACTGATGTGAATACTGGAAAACCAGTATCTCTCTCTAGTTCTTATTTTCACAGAGAGAAATCGAATATTTTCAGTCCACAGGAATTGCCAGGTAGTCATGTAACTGAAGATGTGCTGAAGGTTTCAACAATTCCTGGACCAGCTGGCCAGAAAACAGTATTACCAACAGCTCTTCCTAGTTCCTTTTCACATCGAGAGAAACCAGATATTTTCTATCAAAAGGATTTGCCAGATAGACATCTAACTGAAGATGCTCTAAAGATCTCAAGTGCTCTTGGGCAAGCTGATCAAATTACCGGATTACAAACAGTTCCCTCTGGTACTTACTCACATGGTGAGAATCACAAGCTTGTTTCAGAACATGTCCAAAGGCTAATAGATAATTTGAATTCTTCTGACTCCAGTGTTAGCTCAAATAATGTGCTTTTAAATTCTCAGGCTGATGACAGAGTTGTAATAAATAAACCAGAATCTGCAGGTTTTAGAGATGTTGGCTCTGAAGAAATCCAGGATGCAGAAAATAGTGCTAAAACTCTTAAGGAAATTCGGACACTTTTGATGGAGGCAGAAAATATGGCACTGAAACGATGCAATTTTCCTGCTCCCCTTGCCCGTTTCAGAGATATTAGTGATATTTCATTTATACAATCTAAGAAGGTGGTTTGCTTCAAAGAACCCTCTTCCACGGGTGTATCTAATGGTGATTTGCTTCACAGACAGCCATTCACAGAGGAAAGCCCAAGCAGCAGGTGCATACAGAAGGATATTGGCACACAGACGAATTTGAAATGCCGGAGAGGCATTGAAAATTGGGAGTTTATTAGTTCAACTACAGTTAGAAGTCCTCTACAGGAAGCAGAGAGCAAAGTCAGTATGGCATTAGAAGAAACTCTTAGGCAATATCAAGCAGCCAAATCTGTAATGAGGTCTGAACCTGAAGGGTGTAGTGGAACCATTGGGAATAAAATTATTATCCCTATGATGACTGTCATAAAAAGTGATTCAAGTAGTGATGCCAGTGATGGAAATGGTTCCTGCTCGTGGGACAGTAATTTACCAGAGTCTTTGGAATCAGTTTCTGATGTTCTTCTAAACTTCTTTCCATATGTTTCACCCAAGACAAGTATAACAGATAGCAGGGAGGAAGAGGGTGTGTCAGAGAGTGAGGATGGTGGTGGTAGCAGTGTAGATTCACTGGCTGCACATGTGAAAAACCTTCTGCAATGTGAATCCTCACTGAATCATGCTAAAGAAATACTCAGAAATGCAGAGGAAGAGGAAAGCCGGGTACGAGCACATGCCTGGAATATGAAGTTCAATTTAGCACATGATTGTGGATACTCCATTTCAGAATTAAATGAAGATGACAGGAGGAAAGTAGAAGAGATCAAGGCAGAGTTATTTGGTCATGGAAGAACAACTGACTTGTCCAAGGGTTTACAGAGTCCACGGGGAATGGGATGCAAGCCAGAAGCTGTATGTAGTCACATTATTATTGAGAGCCATGAAAAGGGATGTTTCCGGACTCTAACTTCTGAACATCCACAACTAGATAGACACCCTTGTGCTTTCAGATCTGCTGGACCCTCAGAAATGACCAGAGGACGGCAGAACCCATCATCATGCAGAGCCAAGCATGTCAACCTTTCTGCATCCTTAGACCAGAACAACTCCCATTTCAAAGTTTGGAATTCCTTGCAGTTAAAAAGTCATTCCCCATTTCAGAACTTTATACCTGATGAATTCAAAATCAGCAAAGGTCTTCGAATGCCATTCGATGAAAAGATGGACCCTTGGCTGTCAGAATTAGTAGAACCTGCTTTTGTGCCACCTAAAGAAGTGGATTTTCATTCTTCATCACAAATGCCGTCCCCAGAACCCATGAAAAAGTTTACTACCTCCATCACTTTTTCATCTCACCGACATTCTAAATGCATTTCCAATTCCTCTGTTGTTAAGGTTGGTGTTACTGAAGGTAGCCAGTGTACTGGAGCATCTGTGGGGGTATTTAATTCTCATTTCACTGAAGAACAAAATCCTCCCAGAGATCTTAAACAGAAAACCTCTTCCCCTTCATCATTTAAAATGCATAGTAATTCACAAGATAAAGAAGTGACTATTTTAGCAGAAGGTAGAAGGCAAAGCCAAAAATTACCTGTTGATTTTGAGCGTTCTTTTCAAGAAGAAAAACCCTTAGAAAGATCAGATTTTACAGGCAGTCATTCTGAGCCCAGTACCAGGGCAAATTGTAGCAATTTCAAGGAAATTCAGATTTCTGATAACCATACCCTTATTAGCATGGGCAGACCAAGTTCCACCCTAGGAGTAAACAGATCGAGTTCCAGACTAGGAGTAAAAGAGAAGAATGTAACTATAACTCCAGATCTTCCTTCTTGCATTTTTCTTGAACAACGAGAGCTCTTTGAACAAAGCAAAGCCCCACGTGCAGATGACCATGTGAGGAAACACCATTCTCCCTCTCCTCAACATCAGGATTATGTAGCTCCAGACCTTCCTTCTTGCATTTTTCTTGAACAACGAGAACTCTTTGAACAGTGCAAAGCCCCATATGTAGATCATCAAATGAGAGAAAACCATTCTCCCCTTCCTCAAGGTCAGGATTCTATAGCTTCAGACCTTCCGTCTCCCATTTCTCTTGAACAATGCCAAAGCAAAGCGCCAGGTGTAGATGACCAAATGAATAAACACCATTTTCCCCTTCCTCAAGGTCAGGATTGTGTAGTGGAAAAGAATAATCAACATAAGCCTAAATCACACATTTCTAATATAAATGTTGAAGCCAAGTTCAATACTGTGGTCTCCCAGTCAGCCCCAAATCACTGTACATTAGCAGCATCTGCATCTACTCCTCCTTCAAATAGAAAAGCACTTTCTTGTGTTCATATAACTCTTTGTCCCAAGACTTCTTCCAAGTTGGATAGTGGAACTTTAGATGAAAGATTCCATTCATTGGATGCTGCTTCTAAAGCGAGGATGAATAGTGAGTTTAACTTTGACTTACATACTGTATCTTCGAGATCACTGGAACCAACCTCCAAATTATTGACCAGTAAACCTGTAGCACAGGATCAAGAATCTTTAGGTTTTCTAGGACCTAAATCTTCACTGGATTTCCAAGTCGTACAGCCTTCTCTTCCAGACAGTAACACTATTACTCAGGACTTGAAAACCATACCTTCTCAGAATAGCCAGATAGTAACCTCCAGGCAAATACAAGTGAACATTTCAGATTTCGAAGGACATTCCAATCCAGAGGGGACCCCAGTATTTGCAGATC**G**ATTACCAGAGAAGATGAAGACCCCACTTTCTGCTTTCTCTGAAAAATTGTCATCTGATGCAGTCACTCAGATAACAACAGAAAGTCCAGAAAAGACCCTATTTTCATCTGAGATTTTTATTAATGCTGAAGATCGTGGACATGAAATTATAGAGCCTGGTAACCAGAAGCTACGCAAAGCTCCTGTCAAGTTTGCCTCATCATCTTCAGTCCAACAGGTTACTTTTTCTCGCGGCACAGATGGCCAGCCTTTATTATTGCCATATAAGCCTTCTGGTAGTACCAAGATGTATTATGTTCCACAATTAAGACAAATTCCTCCATCTCCGGATTCCAAATCAGATACCACCGTTGAAAGCTCCCATTCAGGATCCAATGATGCCATTGCTCCAGACTTCCCAGCTCAGGTGCTAGGCACAAGAGATGATGACCTCTCAGCCACTGTTAACATTAAACATAAAGAAGGAATCTACAGTAAGAGGGTAGTGACTAAGGCATCCTTGCCAGTGGGAGAAAAACCCTTGCAGAATGAAAATGCAGATGCCTCAGTTCAAGTGCTAATCACTGGGGATGAGAACCTCTCAGACAAAAAACAGCAAGAGATTCACAGTACAAGGGCAGTGACTGAGGCTGCCCAGGCTAAAGAAAAAGAATCTTTGCAGAAAGATACTGCAGATTCCAGTGCTGCTGCTGCTGCAGAGCACTCAGCTCAAGTAGGAGACCCAGAAATGAAGAACTTGCCAGACACTAAAGCCATTACACAGAAAGAGGAGATCCATAGGAAGAAGACAGTTCCCGAGGAAGCCTGGCCAAACAATAAAGAATCCCTACAGATCAATATTGAAGAGTCCGAATGTCATTCAGAATTTGAAAATACTACCCGTTCTGTCTTCAGGTCAGCAAAGTTTTACATTCATCATCCCGTACACCTACCAAGTGATCAAGATATTTGCCATGAATCTTTGGGAAAGAGTGTTTTCATGAGACATTCTTGGAAAGATTTCTTTCAGCATCATCCAGACAAACATAGAGAACACATGTGTCTTCCTCTTCCTTATCAAAACATGGACAAGACTAAGACAGATTATACCAGAATAAAGAGCCTCAGCATCAATGTGAATTTGGGAAACAAAGAAGTGATGGATACTACTAAAAGTCAAGTTAGAGATTATCCAAAACATAATGGACAAATTAGTGATCCACAAAGGGATCAGAAGGTCACCCCAGAGCAAACAACTCAGCACACTGTGAGTTTGAATGAACTGTGGAACAAGTATCGGGAGCGACAGAGGCAACAGAGACAGCCTGAGTTGGGTGACAGGAAAGAACTGTCCTTGGTGGACCGACTTGATCGTTTGGCTAAAATTCTTCAGAATCCAATCACACATTCTCTCCAGGTCTCAGAAAGTACACATGATGATAGCAGAGGGGAACGAAGTGTGAAGGAATGGAGTGGTAGACAACAGCAGAGAAATAAGCTTCAGAAAAAGAAGCGGTTTAAAAGCCTAGAGAAAAGCCATAAAAATACAGGCGAGCTTAAAAAAAGCAAGGTGCTTTCTCATCATCGAGCTGGGAGGTCTAATCAAATTAAAATTGAACAGATTAAATTTGATAAATATATTCTGAGTAAACAGCCAGGTTTTAATTATATAAGCAACACTTCTTCGGATTGTCGGCCCTCAGAGGAGAGTGAGCTGCTCACAGATACTACCACCAACATCCTTTCCGGCACCACTTCTACTGTCGAATCAGATATATTGACCCAAACAGATAGAGAGGTGGCTCTGCACGAAAGGAGTAGCTCTGTTTCCACTATTGACACTGCCCGGCTGATTCAAGCTTTTGGCCATGAAAGAGTATGCTTGTCACCCAGACGAATTAAATTATATAGCAGCATCACCAACCAACAGAGGAGATACCTTGAGAAGCGGAGCAAACACAGCAAGAAAGTGCTGAATACAGGTCATCCCCTAGTGACTTCTGAGCACACCAGAAGGAGACACATCCAGGTAGCAAACCATGTGATTTCTTCTGACTCTATTTCCTCTTCTGCCAGTAGTTTCCTGAGCTCAAACTCTACTTTTTGCAACAAGCAGAATGTACACATGTTAAACAAGGGCATACAAGCAGGTAACTTGGAGATTGTGAACGGTGCCAAAAAACACACTCGAGATGTTGGGATAACTTTCCCAACTCCAAGTTCCAGCGAGGCTAAATTGGAAGAGAACAGTGATGTGACTTCTTGGTCAGAAGAAAAACGTGAAGAGAAAATGCTCTTTACCGGTTATCCTGAGGACAGAAAGTTAAAAAAGAACAAGAAGAATTCCCATGAAGGAGTTTCCTGGTTTGTTCCTGTGGAAAATGTGGAGTCTAGATCAAAGAAGGAAAACGTGCCTAACACTTGTGGCCCTGGCATCTCCTGGTTTGAACCAATAACCAAGACCAGACCCTGGAGGGAGCCACTGCGGGAGCAGAACTGTCAGGGGCAGCACCTGGACGGTCGGGGCTACCTGGCAGGCCCAGGCAGAGAGGCTGGCAGAGACCTACTGAGGCCATTTGTGAGAGCAACCCTTCAGGAATCGCTTCAGTTTCACAGACCTGACTTCATCTCCCGCTCTGGGGAGCGGATAAAGCGCCTGAAGTTAATAGTCCAGGAGAGGAAGCTGCAGAGCATGTTACAGACCGAGCGGGATGCACTATTCAACATTGACAGGGAACGGCAGGGCCACCAGAATCGCATGTGCCCGCTGCCCAAGAGAGTCTTCCTGGCTATCCAGAAGAACAAGCCTATCAGCAAGAAGGAAATGATTCAGAGGTCCAAACGGATTTATGAGCAGCTTCCAGAAGTACAGAAAAAGAGAGAAGAAGAGAAGAGAAAATCAGAATATAAGTCATACCGGCTGCGAGCCCAGCTATATAAAAAGAGAGTGACCAATCAACTTCTGGGGAGAAAAGTTCCCTGGGACTGACACAAGTTTATTTTCCTCAGAGCCTTGGAATTCTATTTTATGAACCTAGAGAAGCAGAATCCTTACTTTTGTGAGTCTGGTTGAATAAAGCTTATTCTTTGTCCATGTGTATTTTAGAAATAGTAACTTCTAAAGAGTCTGGAACAAAGTGGTGATTAAAATTCCTAATGGTTTGGGAGCAATACTTTCTGCATAGTGGCCTTGTCCAATGGCCTGTGTGTTACAATGATATGATCATTTCTCAAGAATAAGTCCCTTTTTGTATGTGTTTTTATACTTTTAGAAAATAAAAACTTTAGATTAACTC

**Mutation-DNA sequencing data：**

AGGCGGGCGGCACTGCGCCTAAGCTGGGCCACAACCGCCAGTCAGGGCTCTCCCCTTCCCCTCCCTCCCCCCCTCCTCCTCCTCCTCTGCCGCCCAGAGCGAGACACCAACATGGAGCCCGAGGATCTGCCATGGCCGGGCGAGCTGGAGGAGGAGGAGGAGGAGGAGGAGGAGGAGGAGGAGGAGGAAGAGGAGGAGGCTGCAGCGGCGGCGGCGGCGAACGTGGACGACGTAGTGGTCGTGGAGGAGGTGGAGGAAGAGGCGGGGCGGGAGTTGGACTCCGACTCTCACTACGGGCCCCAGCATCTGGAAAGTATAGACGACGAGGAGGACGAGGAGGCCAAGGCCTGGCTGCAGGCGCACCCCGGCAGGATTTTGCCTCCGCTGTCGCCCCCGCAGCACCGCTACTCGGAGGGCGAGCGGACCTCCCTGGAGAAGATTGTTCCATTGACCTGTCATGTATGGCAACAGATAGTATATCAAGGCAATAGTAGAACACAAATTTCTGATACTAATGTGGTCTGTTTGGAAACAACAGCTCAGCGGGGTTCTGGGGATGATCAGAAAACAGAATCTTGGCATTGTCTTCCTCAAGAAATGGACTCTTCCCAAACCTTGGATACATCCCAGACTAGGTTTAATGTGAGAACGGAAGATACTGAAGTGACAGACTTCCCCTCTCTGGAGGAGGGCATATTGACGCAATCAGAAAATCAAGTAAAGGAACCCAACAGAGATCTCTTCTGTTCTCCACTGCTAGTCATACAAGATAGCTTTGCTTCTCCTGATTTGCCTTTGCTGACCTGTTTGACACAAGACCAAGAATTTGCGCCTGATTCTTTATTTCATCAAAGTGAACTAAGTTTTGCACCTCTGAGGGGAATTCCTGATAAGTCTGAAGATACTGAATGGTCTTCTCGACCATCGGAAGTTAGTGAAGCTTTATTCCAGGCTACTGCAGAAGTAGCTTCAGACTTAGCAAGCAGTCGCTTTAGTGTATCTCAGCACCCGCTTATAGGCAGCACAGCTGTTGGGTCTCAGTGCCCTTTTTTACCTTCTGAACAAGGGAATAATGAAGAGACTATTTCGTCTGTTGATGAACTGAAAATTCCCAAAGACTGTGATCGTTATGATGATCTTTGTTCATATATGTCATGGAAGACACGAAAAGATACACAGTGGCCTGAAAACAATTTAGCTGATAAAGATCAAGTTTCAGTTGCAACTTCATTTGACATAACTGATGAAAACATAGCTACTAAAAGAAGTGACCATTTTGATGCTGCTCGTTCATATGGGCAGTATTGGACACAGGAAGATTCATCTAAGCAGGCAGAAACATATTTAACCAAGGGCCTGCAGGGGAAGGTTGAGTCTGACGTCATTACTCTGGATGGCCTAAATGAAAATGCTGTTGTATGCAGTGAAAGAGTTGCTGAACTACAAAGAAAGCCAACAAGAGAGTCGGAATATCACTCTTCAGATCTCAGAATGTTGAGGATGTCTCCTGACACTGTGCCAAAGGCTCCTAAACATTTAAAAGCAGGAGACACTTCTAAAGGAGGCATAGCTAAAGTTACTCAATCCAACTTGAAGTCAGGCATCACTACCACTCCTGTTGATTCAGACATTGGATCTCATTTATCCTTGTCCCTTGAGGACCTGTCTCAGTTGGCTGTAAGTTCTCCTCTAGAAACTACTACTGGTCAACACACTGATACTCTCAACCAAAAGACATTAGCAGATACTCATCTAACTGAAGAGACTCTGAAAGTCACAGCTATTCCTGAACCAGCTGACCAGAAGACTGCAACACCAACAGTACTCTCTAGTTCCCACTCACATAGGGGGAAGCCCAGCATTTTCTACCAGCAGGGCTTGCCAGACAGTCATCTAACTGAAGAGGCTTTGAAAGTTTCAGCTGCTCCTGGACTAGCTGACCAGACAACTGGCATGTCAACTCTAACCTCTACTTCCTACTCACATAGAGAGAAGCCTGGTACTTTTTACCAACAAGAGTTACCAGAGAGTAACTTAACCGAAGAGCCTTTGGAAGTTTCAGCTGCTCCTGGCCCAGTGGAGCAGAAGACGGGAATACCTACAGTATCCTCTACATCCCACTCACATGTAGAGGACCTCCTCTTTTTCTATCGACAGACCTTGCCAGATGGTCATCTAACTGATCAGGCTCTGAAAGTCTCAGCTGTGTCTGGACCAGCTGACCAGAAGACTGGGACAGCAACAGTACTCTCTACTCCCCACTCACATAGAGAGAAGCCTGGTATTTTTTACCAACAAGAGTTCGCAGACAGTCATCAAACTGAAGAGACTCTTACTAAAGTTTCAGCCACTCCTGGACCAGCTGACCAGAAGACTGAGATACCAGCAGTACAGTCTAGTTCTTACTCACAAAGAGAAAAGCCTAGTATTTTGTACCCACAGGACTTAGCAGACAGTCATCTACCTGAAGAGGGTCTGAAAGTTTCAGCTGTTGCTGGACCAGCTGACCAGAAGACTGGCCTACCAACAGTACCCTCTAGTGCATACTCACACAGAGAGAAGCTCCTTGTTTTCTACCAACAGGCCTTGCTGGACAGCCATCTACCCGAAGAGGCTCTGAAAGTTTCAGCTGTTTCTGGACCAGCTGACGGAAAGACTGGGACACCAGCTGTAACCTCTACTTCCTCTGCGTCCTCTTCACTTGGAGAAAAGCCCAGTGCTTTCTATCAGCAGACCTTACCCAATAGTCATCTAACTGAAGAGGCTCTGAAAGTATCAATTGTTCCTGGACCAGGTGATCAGAAGACTGGGATACCCTCAGCACCATCTAGTTTCTACTCACACAGAGAGAAGCCCATTATTTTTTCCCAGCAGACCCTGCCAGACTTTCTTTTCCCTGAAGAAGCTCTGAAGGTTTCAGCTGTTTCTGTATTGGCTGCCCAGAAGACTGGGACACCAACAGTGTCCTCTAATTCTCACTCACATAGCGAGAAATCTAGTGTTTTCTACCAGCAAGAGTTGCCAGACAGTGATCTACCTAGAGAATCTCTGAAAATGTCTGCTATTCCTGGACTGACTGACCAGAAGACTGTCCCAACACCAACAGTACCTTCAGGTTCCTTCTCACATAGAGAGAAGCCCAGTATTTTCTATCAACAGGAGTGGCCAGATAGTTATGCAACTGAAAAGGCTCTGAAAGTTTCAACTGGCCCTGGACCAGCTGACCAGAAGACTGAGATACCAGCAGTACAGTCTAGTTCTTACCCACAGAGGGAGAAGCCTAGTGTTTTGTACCCACAGGTGTTATCAGACAGTCATCTACCTGAAGAGAGTCTGAAAGTTTCAGCCTTCCCTGGACCAGCTGACCAGATGACTGACACACCAGCAGTACCGTCTACTTTCTACTCACAAAGAGAGAAGCCTGGTATTTTCTACCAACAGACCTTGCCAGAGAGTCATCTGCCTAAAGAGGCTCTGAAAATTTCAGTAGCTCCTGGACTAGCAGACCAGAAGACTGGCACACCAACTGTAACCTCAACTTCCTACTCACAACATAGAGAAAAGCCCAGCATTTTCCACCAGCAGGCCTTGCCAGGTACTCATATACCTGAAGAGGCTCAGAAAGTTTCAGCTGTTACTGGACCAGGTAACCAGAAGACTTGGATACCAAGAGTACTTTCTACCTTCTACTCACAAAGAGAGAAACCTGGTATTTTCTATCAACAGACCTTGCCAGGTAGTCACATACCTGAAGAGGCACAGAAAGTTTCACCTGTTCTTGGACCAGCTGACCAGAAGACTGGGACACCAACTCCAACCTCTGCTTCTTACTCACACACAGAGAAGCCTGGTATTTTCTACCAACAGGTCTTGCCAGATAATCATCCAACTGAAGAGGCTCTGAAAATTTCAGTTGCCTCTGAACCAGTTGACCAGACAACTGGCACACCAGCTGTAACCTCTACTTCCTACTCACAATATAGAGAGAAGCCCAGCATTTTCTACCAACAGTCGTTGCCAAGTAGTCATCTAACTGAAGAGGCTAAGAATGTTTCAGCGGTTCCTGGACCAGCTGACCAGAAGACTGTGATACCAATTTTACCCTCTACTTTCTACTCACACACAGAGAAGCCTGGTGTTTTCTACCAACAGGTCTTGCCACATAGTCATCCAACTGAAGAGGCTCTGAAAATTTCAGTTGCCTCTGAACCAGTTGACCAGACAACTGGCACACCAACTGTAACCTCTACTTCTTACTCACAACATACAGAGAAGCCGAGTATTTTCTACCAACAGTCGTTGCCAGGTAGTCATCTAACTGAAGAGGCTAAGAACGTTTCAGCGGTTCCTGGACCAGGTGACCGGAAGACTGGGATACCAACTTTACCCTCTACTTTCTACTCACACACAGAGAAGCCTGGTAGTTTCTACCAACAGGTCTTGCCACATAGTCATCTACCTGAAGAGGCTTTGGAAGTTTCAGTTGCTCCTGGACCAGTTGACCAGACGATTGGCACACCAACTGTAACCTCCCCTTCCAGCTCATTTGGAGAGAAGCCCATTGTTATCTACAAACAGGCCTTTCCAGAGGGTCATCTACCTGAAGAGTCTCTGAAAGTTTCAGTTGCTCCTGGACCAGTTGGCCAGACAACTGGCGCACCAACTATAACCTCTCCTTCCTACTCACAACATAGAGCAAAGTCTGGCAGTTTCTACCAACTGGCATTGCTAGGTAGTCAAATACCTGAAGAGGCTCTCAGAGTTTCTTCTGCTCCTGGACCAGCTGACCAGACAACTGGCATACCAACCATAACCTCTACTTCCTACTCATTTGGAGAGAAGCCGATTGTTAACTACAAACAGGCCTTTCCAGATGGTCATCTACCTGAAGAGGCTCTGAAAGTTTCCATTGTTTCTGGACCTACTGAAAAAAAGACTGACATACCAGCAGGACCTTTAGGTTCCAGTGCACTTGGAGAGAAGCCCATTACTTTCTACCGGCAGGCTCTGCTAGACAGTCCTCTAAATAAAGAGGTTGTGAAAGTTTCAGCTGCTCCTGGACCAGCTGACCAGAAGACTGAGACATTACCAGTACATTCTACTAGCTACTCAAATAGGGGGAAGCCTGTCATTTTCTACCAGCAGACCCTATCAGACAGTCATTTACCTGAAGAAGCTCTGAAAGTTCCACCTGTTCCTGGACCAGATGCCCAGAAGACTGAGACACCATCAGTATCCTCTAGTTTATACTCATATAGAGAGAAGCCCATTGTCTTCTACCAACAGGCCCTGCCAGACAGTGAGCTAACTCAAGAAGCTCTGAAAGTTTCAGCTGTTCCTCAACCAGCTGACCAGAAGACTGGGTTATCTACTGTAACTTCCTCTTTCTATTCACATACAGAGAAGCCTAATATTTCTTACCAGCAAGAGTTGCCAGATAGTCATCTAACTGAAGAGGCTCTGAAAGTTTCAAATGTTCCTGGACCAGCTGACCAGAAGACTGGGGTATCAACAGTAACCTCTACTTCCTACTCACACAGAGAGAAGCCCATTGTTTCCTACCAGCGAGAGTTGCCGCATTTTACTGAAGCAGGTTTGAAAATTTTAAGAGTTCCTGGACCAGCTGACCAGAAGACTGGAATAAACATCCTGCCCTCTAATTCCTACCCACAGAGAGAGCACTCTGTCATTTCTTATGAGCAGGAGTTGCCAGATCTTACTGAAGTAACTTTGAAAGCAATAGGGGTTCCTGGGCCTGCTGACCAGAAGACTGGGATACAAATAGCATCCTCTAGTTCCTACTCAAATAGAGAGAAGGCCAGTATTTTTCATCAGCAGGAGTTGCCAGATGTTACTGAAGAAGCTTTAAATGTTTTTGTTGTTCCTGGACAAGGTGACCGGAAGACTGAGATACCAACAGTACCTTTAAGTTACTACTCACGTAGAGAGAAGCCCAGTGTTATCTCTCAACAGGAGTTGCCAGACAGTCATCTCACAGAAGAGGCTCTGAAAGTTTCACCTGTTTCTATACCAGCAGAGCAGAAGACTGGGATACCAATAGGACTGTCTAGTTCCTACTCACATTCACATAAAGAGAAACTCAAGATTTCAACTGTGCATATACCAGATGACCAGAAAACTGAGTTTCCAGCAGCTACCCTTAGTTCCTACTCACAAATAGAGAAGCCCAAGATTTCAACTGTGATTGGACCAAATGACCAGAAGACTCCATCCCAGACAGCTTTTCATAGTTCCTATTCTCAAACAGTAAAGCCCAATATTTTATTTCAACAGCAGTTGCCAGATAGAGATCAAAGTAAAGGTATTCTAAAGATTTCAGCTGTCCCTGAACTAACTGATGTGAATACTGGAAAACCAGTATCTCTCTCTAGTTCTTATTTTCACAGAGAGAAATCGAATATTTTCAGTCCACAGGAATTGCCAGGTAGTCATGTAACTGAAGATGTGCTGAAGGTTTCAACAATTCCTGGACCAGCTGGCCAGAAAACAGTATTACCAACAGCTCTTCCTAGTTCCTTTTCACATCGAGAGAAACCAGATATTTTCTATCAAAAGGATTTGCCAGATAGACATCTAACTGAAGATGCTCTAAAGATCTCAAGTGCTCTTGGGCAAGCTGATCAAATTACCGGATTACAAACAGTTCCCTCTGGTACTTACTCACATGGTGAGAATCACAAGCTTGTTTCAGAACATGTCCAAAGGCTAATAGATAATTTGAATTCTTCTGACTCCAGTGTTAGCTCAAATAATGTGCTTTTAAATTCTCAGGCTGATGACAGAGTTGTAATAAATAAACCAGAATCTGCAGGTTTTAGAGATGTTGGCTCTGAAGAAATCCAGGATGCAGAAAATAGTGCTAAAACTCTTAAGGAAATTCGGACACTTTTGATGGAGGCAGAAAATATGGCACTGAAACGATGCAATTTTCCTGCTCCCCTTGCCCGTTTCAGAGATATTAGTGATATTTCATTTATACAATCTAAGAAGGTGGTTTGCTTCAAAGAACCCTCTTCCACGGGTGTATCTAATGGTGATTTGCTTCACAGACAGCCATTCACAGAGGAAAGCCCAAGCAGCAGGTGCATACAGAAGGATATTGGCACACAGACGAATTTGAAATGCCGGAGAGGCATTGAAAATTGGGAGTTTATTAGTTCAACTACAGTTAGAAGTCCTCTACAGGAAGCAGAGAGCAAAGTCAGTATGGCATTAGAAGAAACTCTTAGGCAATATCAAGCAGCCAAATCTGTAATGAGGTCTGAACCTGAAGGGTGTAGTGGAACCATTGGGAATAAAATTATTATCCCTATGATGACTGTCATAAAAAGTGATTCAAGTAGTGATGCCAGTGATGGAAATGGTTCCTGCTCGTGGGACAGTAATTTACCAGAGTCTTTGGAATCAGTTTCTGATGTTCTTCTAAACTTCTTTCCATATGTTTCACCCAAGACAAGTATAACAGATAGCAGGGAGGAAGAGGGTGTGTCAGAGAGTGAGGATGGTGGTGGTAGCAGTGTAGATTCACTGGCTGCACATGTGAAAAACCTTCTGCAATGTGAATCCTCACTGAATCATGCTAAAGAAATACTCAGAAATGCAGAGGAAGAGGAAAGCCGGGTACGAGCACATGCCTGGAATATGAAGTTCAATTTAGCACATGATTGTGGATACTCCATTTCAGAATTAAATGAAGATGACAGGAGGAAAGTAGAAGAGATCAAGGCAGAGTTATTTGGTCATGGAAGAACAACTGACTTGTCCAAGGGTTTACAGAGTCCACGGGGAATGGGATGCAAGCCAGAAGCTGTATGTAGTCACATTATTATTGAGAGCCATGAAAAGGGATGTTTCCGGACTCTAACTTCTGAACATCCACAACTAGATAGACACCCTTGTGCTTTCAGATCTGCTGGACCCTCAGAAATGACCAGAGGACGGCAGAACCCATCATCATGCAGAGCCAAGCATGTCAACCTTTCTGCATCCTTAGACCAGAACAACTCCCATTTCAAAGTTTGGAATTCCTTGCAGTTAAAAAGTCATTCCCCATTTCAGAACTTTATACCTGATGAATTCAAAATCAGCAAAGGTCTTCGAATGCCATTCGATGAAAAGATGGACCCTTGGCTGTCAGAATTAGTAGAACCTGCTTTTGTGCCACCTAAAGAAGTGGATTTTCATTCTTCATCACAAATGCCGTCCCCAGAACCCATGAAAAAGTTTACTACCTCCATCACTTTTTCATCTCACCGACATTCTAAATGCATTTCCAATTCCTCTGTTGTTAAGGTTGGTGTTACTGAAGGTAGCCAGTGTACTGGAGCATCTGTGGGGGTATTTAATTCTCATTTCACTGAAGAACAAAATCCTCCCAGAGATCTTAAACAGAAAACCTCTTCCCCTTCATCATTTAAAATGCATAGTAATTCACAAGATAAAGAAGTGACTATTTTAGCAGAAGGTAGAAGGCAAAGCCAAAAATTACCTGTTGATTTTGAGCGTTCTTTTCAAGAAGAAAAACCCTTAGAAAGATCAGATTTTACAGGCAGTCATTCTGAGCCCAGTACCAGGGCAAATTGTAGCAATTTCAAGGAAATTCAGATTTCTGATAACCATACCCTTATTAGCATGGGCAGACCAAGTTCCACCCTAGGAGTAAACAGATCGAGTTCCAGACTAGGAGTAAAAGAGAAGAATGTAACTATAACTCCAGATCTTCCTTCTTGCATTTTTCTTGAACAACGAGAGCTCTTTGAACAAAGCAAAGCCCCACGTGCAGATGACCATGTGAGGAAACACCATTCTCCCTCTCCTCAACATCAGGATTATGTAGCTCCAGACCTTCCTTCTTGCATTTTTCTTGAACAACGAGAACTCTTTGAACAGTGCAAAGCCCCATATGTAGATCATCAAATGAGAGAAAACCATTCTCCCCTTCCTCAAGGTCAGGATTCTATAGCTTCAGACCTTCCGTCTCCCATTTCTCTTGAACAATGCCAAAGCAAAGCGCCAGGTGTAGATGACCAAATGAATAAACACCATTTTCCCCTTCCTCAAGGTCAGGATTGTGTAGTGGAAAAGAATAATCAACATAAGCCTAAATCACACATTTCTAATATAAATGTTGAAGCCAAGTTCAATACTGTGGTCTCCCAGTCAGCCCCAAATCACTGTACATTAGCAGCATCTGCATCTACTCCTCCTTCAAATAGAAAAGCACTTTCTTGTGTTCATATAACTCTTTGTCCCAAGACTTCTTCCAAGTTGGATAGTGGAACTTTAGATGAAAGATTCCATTCATTGGATGCTGCTTCTAAAGCGAGGATGAATAGTGAGTTTAACTTTGACTTACATACTGTATCTTCGAGATCACTGGAACCAACCTCCAAATTATTGACCAGTAAACCTGTAGCACAGGATCAAGAATCTTTAGGTTTTCTAGGACCTAAATCTTCACTGGATTTCCAAGTCGTACAGCCTTCTCTTCCAGACAGTAACACTATTACTCAGGACTTGAAAACCATACCTTCTCAGAATAGCCAGATAGTAACCTCCAGGCAAATACAAGTGAACATTTCAGATTTCGAAGGACATTCCAATCCAGAGGGGACCCCAGTATTTGCAGATC**A**ATTACCAGAGAAGATGAAGACCCCACTTTCTGCTTTCTCTGAAAAATTGTCATCTGATGCAGTCACTCAGATAACAACAGAAAGTCCAGAAAAGACCCTATTTTCATCTGAGATTTTTATTAATGCTGAAGATCGTGGACATGAAATTATAGAGCCTGGTAACCAGAAGCTACGCAAAGCTCCTGTCAAGTTTGCCTCATCATCTTCAGTCCAACAGGTTACTTTTTCTCGCGGCACAGATGGCCAGCCTTTATTATTGCCATATAAGCCTTCTGGTAGTACCAAGATGTATTATGTTCCACAATTAAGACAAATTCCTCCATCTCCGGATTCCAAATCAGATACCACCGTTGAAAGCTCCCATTCAGGATCCAATGATGCCATTGCTCCAGACTTCCCAGCTCAGGTGCTAGGCACAAGAGATGATGACCTCTCAGCCACTGTTAACATTAAACATAAAGAAGGAATCTACAGTAAGAGGGTAGTGACTAAGGCATCCTTGCCAGTGGGAGAAAAACCCTTGCAGAATGAAAATGCAGATGCCTCAGTTCAAGTGCTAATCACTGGGGATGAGAACCTCTCAGACAAAAAACAGCAAGAGATTCACAGTACAAGGGCAGTGACTGAGGCTGCCCAGGCTAAAGAAAAAGAATCTTTGCAGAAAGATACTGCAGATTCCAGTGCTGCTGCTGCTGCAGAGCACTCAGCTCAAGTAGGAGACCCAGAAATGAAGAACTTGCCAGACACTAAAGCCATTACACAGAAAGAGGAGATCCATAGGAAGAAGACAGTTCCCGAGGAAGCCTGGCCAAACAATAAAGAATCCCTACAGATCAATATTGAAGAGTCCGAATGTCATTCAGAATTTGAAAATACTACCCGTTCTGTCTTCAGGTCAGCAAAGTTTTACATTCATCATCCCGTACACCTACCAAGTGATCAAGATATTTGCCATGAATCTTTGGGAAAGAGTGTTTTCATGAGACATTCTTGGAAAGATTTCTTTCAGCATCATCCAGACAAACATAGAGAACACATGTGTCTTCCTCTTCCTTATCAAAACATGGACAAGACTAAGACAGATTATACCAGAATAAAGAGCCTCAGCATCAATGTGAATTTGGGAAACAAAGAAGTGATGGATACTACTAAAAGTCAAGTTAGAGATTATCCAAAACATAATGGACAAATTAGTGATCCACAAAGGGATCAGAAGGTCACCCCAGAGCAAACAACTCAGCACACTGTGAGTTTGAATGAACTGTGGAACAAGTATCGGGAGCGACAGAGGCAACAGAGACAGCCTGAGTTGGGTGACAGGAAAGAACTGTCCTTGGTGGACCGACTTGATCGTTTGGCTAAAATTCTTCAGAATCCAATCACACATTCTCTCCAGGTCTCAGAAAGTACACATGATGATAGCAGAGGGGAACGAAGTGTGAAGGAATGGAGTGGTAGACAACAGCAGAGAAATAAGCTTCAGAAAAAGAAGCGGTTTAAAAGCCTAGAGAAAAGCCATAAAAATACAGGCGAGCTTAAAAAAAGCAAGGTGCTTTCTCATCATCGAGCTGGGAGGTCTAATCAAATTAAAATTGAACAGATTAAATTTGATAAATATATTCTGAGTAAACAGCCAGGTTTTAATTATATAAGCAACACTTCTTCGGATTGTCGGCCCTCAGAGGAGAGTGAGCTGCTCACAGATACTACCACCAACATCCTTTCCGGCACCACTTCTACTGTCGAATCAGATATATTGACCCAAACAGATAGAGAGGTGGCTCTGCACGAAAGGAGTAGCTCTGTTTCCACTATTGACACTGCCCGGCTGATTCAAGCTTTTGGCCATGAAAGAGTATGCTTGTCACCCAGACGAATTAAATTATATAGCAGCATCACCAACCAACAGAGGAGATACCTTGAGAAGCGGAGCAAACACAGCAAGAAAGTGCTGAATACAGGTCATCCCCTAGTGACTTCTGAGCACACCAGAAGGAGACACATCCAGGTAGCAAACCATGTGATTTCTTCTGACTCTATTTCCTCTTCTGCCAGTAGTTTCCTGAGCTCAAACTCTACTTTTTGCAACAAGCAGAATGTACACATGTTAAACAAGGGCATACAAGCAGGTAACTTGGAGATTGTGAACGGTGCCAAAAAACACACTCGAGATGTTGGGATAACTTTCCCAACTCCAAGTTCCAGCGAGGCTAAATTGGAAGAGAACAGTGATGTGACTTCTTGGTCAGAAGAAAAACGTGAAGAGAAAATGCTCTTTACCGGTTATCCTGAGGACAGAAAGTTAAAAAAGAACAAGAAGAATTCCCATGAAGGAGTTTCCTGGTTTGTTCCTGTGGAAAATGTGGAGTCTAGATCAAAGAAGGAAAACGTGCCTAACACTTGTGGCCCTGGCATCTCCTGGTTTGAACCAATAACCAAGACCAGACCCTGGAGGGAGCCACTGCGGGAGCAGAACTGTCAGGGGCAGCACCTGGACGGTCGGGGCTACCTGGCAGGCCCAGGCAGAGAGGCTGGCAGAGACCTACTGAGGCCATTTGTGAGAGCAACCCTTCAGGAATCGCTTCAGTTTCACAGACCTGACTTCATCTCCCGCTCTGGGGAGCGGATAAAGCGCCTGAAGTTAATAGTCCAGGAGAGGAAGCTGCAGAGCATGTTACAGACCGAGCGGGATGCACTATTCAACATTGACAGGGAACGGCAGGGCCACCAGAATCGCATGTGCCCGCTGCCCAAGAGAGTCTTCCTGGCTATCCAGAAGAACAAGCCTATCAGCAAGAAGGAAATGATTCAGAGGTCCAAACGGATTTATGAGCAGCTTCCAGAAGTACAGAAAAAGAGAGAAGAAGAGAAGAGAAAATCAGAATATAAGTCATACCGGCTGCGAGCCCAGCTATATAAAAAGAGAGTGACCAATCAACTTCTGGGGAGAAAAGTTCCCTGGGACTGACACAAGTTTATTTTCCTCAGAGCCTTGGAATTCTATTTTATGAACCTAGAGAAGCAGAATCCTTACTTTTGTGAGTCTGGTTGAATAAAGCTTATTCTTTGTCCATGTGTATTTTAGAAATAGTAACTTCTAAAGAGTCTGGAACAAAGTGGTGATTAAAATTCCTAATGGTTTGGGAGCAATACTTTCTGCATAGTGGCCTTGTCCAATGGCCTGTGTGTTACAATGATATGATCATTTCTCAAGAATAAGTCCCTTTTTGTATGTGTTTTTATACTTTTAGAAAATAAAAACTTTAGATTAACTC

**2. Protein sequences:**

**WT-Protein sequences ( Gene : ALMS1, Uniprot ID: Q8TCU4)：**

MEPEDLPWPG ELEEEEEEEE EEEEEEEEAA AAAAANVDDV VVVEEVEEEA GRELDSDSHY
GPQHLESIDD EEDEEAKAWL QAHPGRILPP LSPPQHRYSE GERTSLEKIV PLTCHVWQQI
VYQGNSRTQI SDTNVVCLET TAQRGSGDDQ KTESWHCLPQ EMDSSQTLDT SQTRFNVRTE
DTEVTDFPSL EEGILTQSEN QVKEPNRDLF CSPLLVIQDS FASPDLPLLT CLTQDQEFAP
DSLFHQSELS FAPLRGIPDK SEDTEWSSRP SEVSEALFQA TAEVASDLAS SRFSVSQHPL
IGSTAVGSQC PFLPSEQGNN EETISSVDEL KIPKDCDRYD DLCSYMSWKT RKDTQWPENN
LADKDQVSVA TSFDITDENI ATKRSDHFDA ARSYGQYWTQ EDSSKQAETY LTKGLQGKVE
SDVITLDGLN ENAVVCSERV AELQRKPTRE SEYHSSDLRM LRMSPDTVPK APKHLKAGDT
SKGGIAKVTQ SNLKSGITTT PVDSDIGSHL SLSLEDLSQL AVSSLETTTG QHTDTLNQKT
LADTHLTEET LKVTAIPEPA DQKTATPTVL SSSHSHRGKP SIFYQQGLPD SHLTEEALKV
SAAPGLADQT TGMSTLTSTS YSHREKPGTF YQQELPESNL TEEPLEVSAA PGPVEQKTGI
PTVSSTSHSH VEDLLFFYRQ TLPDGHLTDQ ALKVSAVSGP ADQKTGTATV LSTPHSHREK
PGIFYQQEFA DSHQTEETLT KVSATPGPAD QKTEIPAVQS SSYSQREKPS ILYPQDLADS
HLPEEGLKVS AVAGPADQKT GLPTVPSSAY SHREKLLVFY QQALLDSHLP EEALKVSAVS
GPADGKTGTP AVTSTSSASS SLGEKPSAFY QQTLPNSHLT EEALKVSIVP GPGDQKTGIP
SAPSSFYSHR EKPIIFSQQT LPDFLFPEEA LKVSAVSVLA AQKTGTPTVS SNSHSHSEKS
SVFYQQELPD SDLPRESLKM SAIPGLTDQK TVPTPTVPSG SFSHREKPSI FYQQEWPDSY
ATEKALKVST GPGPADQKTE IPAVQSSSYP QREKPSVLYP QVLSDSHLPE ESLKVSAFPG
PADQMTDTPA VPSTFYSQRE KPGIFYQQTL PESHLPKEAL KISVAPGLAD QKTGTPTVTS
TSYSQHREKP SIFHQQALPG THIPEEAQKV SAVTGPGNQK TWIPRVLSTF YSQREKPGIF
YQQTLPGSHI PEEAQKVSPV LGPADQKTGT PTPTSASYSH TEKPGIFYQQ VLPDNHPTEE
ALKISVASEP VDQTTGTPAV TSTSYSQYRE KPSIFYQQSL PSSHLTEEAK NVSAVPGPAD
QKTVIPILPS TFYSHTEKPG VFYQQVLPHS HPTEEALKIS VASEPVDQTT GTPTVTSTSY
SQHTEKPSIF YQQSLPGSHL TEEAKNVSAV PGPGDRKTGI PTLPSTFYSH TEKPGSFYQQ
VLPHSHLPEE ALEVSVAPGP VDQTIGTPTV TSPSSSFGEK PIVIYKQAFP EGHLPEESLK
VSVAPGPVGQ TTGAPTITSP SYSQHRAKSG SFYQLALLGS QIPEEALRVS SAPGPADQTT
GIPTITSTSY SFGEKPIVNY KQAFPDGHLP EEALKVSIVS GPTEKKTDIP AGPLGSSALG
EKPITFYRQA LLDSPLNKEV VKVSAAPGPA DQKTETLPVH STSYSNRGKP VIFYQQTLSD
SHLPEEALKV PPVPGPDAQK TETPSVSSSL YSYREKPIVF YQQALPDSEL TQEALKVSAV
PQPADQKTGL STVTSSFYSH TEKPNISYQQ ELPDSHLTEE ALKVSNVPGP ADQKTGVSTV
TSTSYSHREK PIVSYQRELP HFTEAGLKIL RVPGPADQKT GINILPSNSY PQREHSVISY
EQELPDLTEV TLKAIGVPGP ADQKTGIQIA SSSSYSNREK ASIFHQQELP DVTEEALNVF
VVPGQGDRKT EIPTVPLSYY SRREKPSVIS QQELPDSHLT EEALKVSPVS IPAEQKTGIP
IGLSSSYSHS HKEKLKISTV HIPDDQKTEF PAATLSSYSQ IEKPKISTVI GPNDQKTPSQ
TAFHSSYSQT VKPNILFQQQ LPDRDQSKGI LKISAVPELT DVNTGKPVSL SSSYFHREKS
NIFSPQELPG SHVTEDVLKV STIPGPAGQK TVLPTALPSS FSHREKPDIF YQKDLPDRHL
TEDALKISSA LGQADQITGL QTVPSGTYSH GENHKLVSEH VQRLIDNLNS SDSSVSSNNV
LLNSQADDRV VINKPESAGF RDVGSEEIQD AENSAKTLKE IRTLLMEAEN MALKRCNFPA
PLARFRDISD ISFIQSKKVV CFKEPSSTGV SNGDLLHRQP FTEESPSSRC IQKDIGTQTN
LKCRRGIENW EFISSTTVRS PLQEAESKVS MALEETLRQY QAAKSVMRSE PEGCSGTIGN
KIIIPMMTVI KSDSSSDASD GNGSCSWDSN LPESLESVSD VLLNFFPYVS PKTSITDSRE
EEGVSESEDG GGSSVDSLAA HVKNLLQCES SLNHAKEILR NAEEEESRVR AHAWNMKFNL
AHDCGYSISE LNEDDRRKVE EIKAELFGHG RTTDLSKGLQ SPRGMGCKPE AVCSHIIIES
HEKGCFRTLT SEHPQLDRHP CAFRSAGPSE MTRGRQNPSS CRAKHVNLSA SLDQNNSHFK
VWNSLQLKSH SPFQNFIPDE FKISKGLRMP FDEKMDPWLS ELVEPAFVPP KEVDFHSSSQ
MPSPEPMKKF TTSITFSSHR HSKCISNSSV VKVGVTEGSQ CTGASVGVFN SHFTEEQNPP
RDLKQKTSSP SSFKMHSNSQ DKEVTILAEG RRQSQKLPVD FERSFQEEKP LERSDFTGSH
SEPSTRANCS NFKEIQISDN HTLISMGRPS STLGVNRSSS RLGVKEKNVT ITPDLPSCIF
LEQRELFEQS KAPRADDHVR KHHSPSPQHQ DYVAPDLPSC IFLEQRELFE QCKAPYVDHQ
MRENHSPLPQ GQDSIASDLP SPISLEQCQS KAPGVDDQMN KHHFPLPQGQ DCVVEKNNQH
KPKSHISNIN VEAKFNTVVS QSAPNHCTLA ASASTPPSNR KALSCVHITL CPKTSSKLDS
GTLDERFHSL DAASKARMNS EFNFDLHTVS SRSLEPTSKL LTSKPVAQDQ ESLGFLGPKS
SLDFQVVQPS LPDSNTITQD LKTIPSQNSQ IVTSRQIQVN ISDFEGHSNP EGTPVFAD**R**L
PEKMKTPLSA FSEKLSSDAV TQITTESPEK TLFSSEIFIN AEDRGHEIIE PGNQKLRKAP
VKFASSSSVQ QVTFSRGTDG QPLLLPYKPS GSTKMYYVPQ LRQIPPSPDS KSDTTVESSH
SGSNDAIAPD FPAQVLGTRD DDLSATVNIK HKEGIYSKRV VTKASLPVGE KPLQNENADA
SVQVLITGDE NLSDKKQQEI HSTRAVTEAA QAKEKESLQK DTADSSAAAA AEHSAQVGDP
EMKNLPDTKA ITQKEEIHRK KTVPEEAWPN NKESLQINIE ESECHSEFEN TTRSVFRSAK
FYIHHPVHLP SDQDICHESL GKSVFMRHSW KDFFQHHPDK HREHMCLPLP YQNMDKTKTD
YTRIKSLSIN VNLGNKEVMD TTKSQVRDYP KHNGQISDPQ RDQKVTPEQT TQHTVSLNEL
WNKYRERQRQ QRQPELGDRK ELSLVDRLDR LAKILQNPIT HSLQVSESTH DDSRGERSVK
EWSGRQQQRN KLQKKKRFKS LEKSHKNTGE LKKSKVLSHH RAGRSNQIKI EQIKFDKYIL
SKQPGFNYIS NTSSDCRPSE ESELLTDTTT NILSGTTSTV ESDILTQTDR EVALHERSSS
VSTIDTARLI QAFGHERVCL SPRRIKLYSS ITNQQRRYLE KRSKHSKKVL NTGHPLVTSE
HTRRRHIQVA NHVISSDSIS SSASSFLSSN STFCNKQNVH MLNKGIQAGN LEIVNGAKKH
TRDVGITFPT PSSSEAKLEE NSDVTSWSEE KREEKMLFTG YPEDRKLKKN KKNSHEGVSW
FVPVENVESR SKKENVPNTC GPGISWFEPI TKTRPWREPL REQNCQGQHL DGRGYLAGPG
REAGRDLLRP FVRATLQESL QFHRPDFISR SGERIKRLKL IVQERKLQSM LQTERDALFN
IDRERQGHQN RMCPLPKRVF LAIQKNKPIS KKEMIQRSKR IYEQLPEVQK KREEEKRKSE
YKSYRLRAQL YKKRVTNQLL GRKVPWD*

**ALMS1 NM_015120 c.9536G>A (p.R3179Q)-Protein sequences：**

MEPEDLPWPG ELEEEEEEEE EEEEEEEEAA AAAAANVDDV VVVEEVEEEA GRELDSDSHY
GPQHLESIDD EEDEEAKAWL QAHPGRILPP LSPPQHRYSE GERTSLEKIV PLTCHVWQQI
VYQGNSRTQI SDTNVVCLET TAQRGSGDDQ KTESWHCLPQ EMDSSQTLDT SQTRFNVRTE
DTEVTDFPSL EEGILTQSEN QVKEPNRDLF CSPLLVIQDS FASPDLPLLT CLTQDQEFAP
DSLFHQSELS FAPLRGIPDK SEDTEWSSRP SEVSEALFQA TAEVASDLAS SRFSVSQHPL
IGSTAVGSQC PFLPSEQGNN EETISSVDEL KIPKDCDRYD DLCSYMSWKT RKDTQWPENN
LADKDQVSVA TSFDITDENI ATKRSDHFDA ARSYGQYWTQ EDSSKQAETY LTKGLQGKVE
SDVITLDGLN ENAVVCSERV AELQRKPTRE SEYHSSDLRM LRMSPDTVPK APKHLKAGDT
SKGGIAKVTQ SNLKSGITTT PVDSDIGSHL SLSLEDLSQL AVSSLETTTG QHTDTLNQKT
LADTHLTEET LKVTAIPEPA DQKTATPTVL SSSHSHRGKP SIFYQQGLPD SHLTEEALKV
SAAPGLADQT TGMSTLTSTS YSHREKPGTF YQQELPESNL TEEPLEVSAA PGPVEQKTGI
PTVSSTSHSH VEDLLFFYRQ TLPDGHLTDQ ALKVSAVSGP ADQKTGTATV LSTPHSHREK
PGIFYQQEFA DSHQTEETLT KVSATPGPAD QKTEIPAVQS SSYSQREKPS ILYPQDLADS
HLPEEGLKVS AVAGPADQKT GLPTVPSSAY SHREKLLVFY QQALLDSHLP EEALKVSAVS
GPADGKTGTP AVTSTSSASS SLGEKPSAFY QQTLPNSHLT EEALKVSIVP GPGDQKTGIP
SAPSSFYSHR EKPIIFSQQT LPDFLFPEEA LKVSAVSVLA AQKTGTPTVS SNSHSHSEKS
SVFYQQELPD SDLPRESLKM SAIPGLTDQK TVPTPTVPSG SFSHREKPSI FYQQEWPDSY
ATEKALKVST GPGPADQKTE IPAVQSSSYP QREKPSVLYP QVLSDSHLPE ESLKVSAFPG
PADQMTDTPA VPSTFYSQRE KPGIFYQQTL PESHLPKEAL KISVAPGLAD QKTGTPTVTS
TSYSQHREKP SIFHQQALPG THIPEEAQKV SAVTGPGNQK TWIPRVLSTF YSQREKPGIF
YQQTLPGSHI PEEAQKVSPV LGPADQKTGT PTPTSASYSH TEKPGIFYQQ VLPDNHPTEE
ALKISVASEP VDQTTGTPAV TSTSYSQYRE KPSIFYQQSL PSSHLTEEAK NVSAVPGPAD
QKTVIPILPS TFYSHTEKPG VFYQQVLPHS HPTEEALKIS VASEPVDQTT GTPTVTSTSY
SQHTEKPSIF YQQSLPGSHL TEEAKNVSAV PGPGDRKTGI PTLPSTFYSH TEKPGSFYQQ
VLPHSHLPEE ALEVSVAPGP VDQTIGTPTV TSPSSSFGEK PIVIYKQAFP EGHLPEESLK
VSVAPGPVGQ TTGAPTITSP SYSQHRAKSG SFYQLALLGS QIPEEALRVS SAPGPADQTT
GIPTITSTSY SFGEKPIVNY KQAFPDGHLP EEALKVSIVS GPTEKKTDIP AGPLGSSALG
EKPITFYRQA LLDSPLNKEV VKVSAAPGPA DQKTETLPVH STSYSNRGKP VIFYQQTLSD
SHLPEEALKV PPVPGPDAQK TETPSVSSSL YSYREKPIVF YQQALPDSEL TQEALKVSAV
PQPADQKTGL STVTSSFYSH TEKPNISYQQ ELPDSHLTEE ALKVSNVPGP ADQKTGVSTV
TSTSYSHREK PIVSYQRELP HFTEAGLKIL RVPGPADQKT GINILPSNSY PQREHSVISY
EQELPDLTEV TLKAIGVPGP ADQKTGIQIA SSSSYSNREK ASIFHQQELP DVTEEALNVF
VVPGQGDRKT EIPTVPLSYY SRREKPSVIS QQELPDSHLT EEALKVSPVS IPAEQKTGIP
IGLSSSYSHS HKEKLKISTV HIPDDQKTEF PAATLSSYSQ IEKPKISTVI GPNDQKTPSQ
TAFHSSYSQT VKPNILFQQQ LPDRDQSKGI LKISAVPELT DVNTGKPVSL SSSYFHREKS
NIFSPQELPG SHVTEDVLKV STIPGPAGQK TVLPTALPSS FSHREKPDIF YQKDLPDRHL
TEDALKISSA LGQADQITGL QTVPSGTYSH GENHKLVSEH VQRLIDNLNS SDSSVSSNNV
LLNSQADDRV VINKPESAGF RDVGSEEIQD AENSAKTLKE IRTLLMEAEN MALKRCNFPA
PLARFRDISD ISFIQSKKVV CFKEPSSTGV SNGDLLHRQP FTEESPSSRC IQKDIGTQTN
LKCRRGIENW EFISSTTVRS PLQEAESKVS MALEETLRQY QAAKSVMRSE PEGCSGTIGN
KIIIPMMTVI KSDSSSDASD GNGSCSWDSN LPESLESVSD VLLNFFPYVS PKTSITDSRE
EEGVSESEDG GGSSVDSLAA HVKNLLQCES SLNHAKEILR NAEEEESRVR AHAWNMKFNL
AHDCGYSISE LNEDDRRKVE EIKAELFGHG RTTDLSKGLQ SPRGMGCKPE AVCSHIIIES
HEKGCFRTLT SEHPQLDRHP CAFRSAGPSE MTRGRQNPSS CRAKHVNLSA SLDQNNSHFK
VWNSLQLKSH SPFQNFIPDE FKISKGLRMP FDEKMDPWLS ELVEPAFVPP KEVDFHSSSQ
MPSPEPMKKF TTSITFSSHR HSKCISNSSV VKVGVTEGSQ CTGASVGVFN SHFTEEQNPP
RDLKQKTSSP SSFKMHSNSQ DKEVTILAEG RRQSQKLPVD FERSFQEEKP LERSDFTGSH
SEPSTRANCS NFKEIQISDN HTLISMGRPS STLGVNRSSS RLGVKEKNVT ITPDLPSCIF
LEQRELFEQS KAPRADDHVR KHHSPSPQHQ DYVAPDLPSC IFLEQRELFE QCKAPYVDHQ
MRENHSPLPQ GQDSIASDLP SPISLEQCQS KAPGVDDQMN KHHFPLPQGQ DCVVEKNNQH
KPKSHISNIN VEAKFNTVVS QSAPNHCTLA ASASTPPSNR KALSCVHITL CPKTSSKLDS
GTLDERFHSL DAASKARMNS EFNFDLHTVS SRSLEPTSKL LTSKPVAQDQ ESLGFLGPKS
SLDFQVVQPS LPDSNTITQD LKTIPSQNSQ IVTSRQIQVN ISDFEGHSNP EGTPVFAD**Q**L
PEKMKTPLSA FSEKLSSDAV TQITTESPEK TLFSSEIFIN AEDRGHEIIE PGNQKLRKAP
VKFASSSSVQ QVTFSRGTDG QPLLLPYKPS GSTKMYYVPQ LRQIPPSPDS KSDTTVESSH
SGSNDAIAPD FPAQVLGTRD DDLSATVNIK HKEGIYSKRV VTKASLPVGE KPLQNENADA
SVQVLITGDE NLSDKKQQEI HSTRAVTEAA QAKEKESLQK DTADSSAAAA AEHSAQVGDP
EMKNLPDTKA ITQKEEIHRK KTVPEEAWPN NKESLQINIE ESECHSEFEN TTRSVFRSAK
FYIHHPVHLP SDQDICHESL GKSVFMRHSW KDFFQHHPDK HREHMCLPLP YQNMDKTKTD
YTRIKSLSIN VNLGNKEVMD TTKSQVRDYP KHNGQISDPQ RDQKVTPEQT TQHTVSLNEL
WNKYRERQRQ QRQPELGDRK ELSLVDRLDR LAKILQNPIT HSLQVSESTH DDSRGERSVK
EWSGRQQQRN KLQKKKRFKS LEKSHKNTGE LKKSKVLSHH RAGRSNQIKI EQIKFDKYIL
SKQPGFNYIS NTSSDCRPSE ESELLTDTTT NILSGTTSTV ESDILTQTDR EVALHERSSS
VSTIDTARLI QAFGHERVCL SPRRIKLYSS ITNQQRRYLE KRSKHSKKVL NTGHPLVTSE
HTRRRHIQVA NHVISSDSIS SSASSFLSSN STFCNKQNVH MLNKGIQAGN LEIVNGAKKH
TRDVGITFPT PSSSEAKLEE NSDVTSWSEE KREEKMLFTG YPEDRKLKKN KKNSHEGVSW
FVPVENVESR SKKENVPNTC GPGISWFEPI TKTRPWREPL REQNCQGQHL DGRGYLAGPG
REAGRDLLRP FVRATLQESL QFHRPDFISR SGERIKRLKL IVQERKLQSM LQTERDALFN
IDRERQGHQN RMCPLPKRVF LAIQKNKPIS KKEMIQRSKR IYEQLPEVQK KREEEKRKSE
YKSYRLRAQL YKKRVTNQLL GRKVPWD*

3.The Results of whole exome sequencing：

http://ns.mygeno.cn:5000/sharing/It2nuyoly

Password：123
